# A Natural Peptide from A Traditional Chinese Medicine Has the Potential to Treat Chronic Atrophic Gastritis by Activating Gastric Stem Cells

**DOI:** 10.1002/advs.202304326

**Published:** 2024-03-27

**Authors:** Ke Li, Xiuying Ma, Zihao Li, Ya Liu, Guiyan Shen, Zecheng Luo, Dong Wang, Li Xia, Zhengting Wang, Ming Tian, Huijuan Liu, Funeng Geng, Baojie Li

**Affiliations:** ^1^ Institute of Traditional Chinese Medicine and Stem Cell Research College of Basic Medical Sciences Chengdu University of Traditional Chinese Medicine Chengdu 611137 China; ^2^ Bio‐X Institutes Shanghai Jiao Tong University Shanghai 200240 China; ^3^ Sichuan Engineering Research Center for Medicinal Animals Sichuan Good Doctor Panxi Pharmaceutical Co., Ltd Chengdu 610000 China; ^4^ Department of Pathophysiology Key Laboratory of Cell Differentiation and Apoptosis of the Chinese Ministry of Education Shanghai Jiao Tong University School of Medicine Shanghai 200025 China; ^5^ Department of Gastroenterology Ruijin Hospital School of Medicine Shanghai Jiao Tong University Shanghai 200025 China; ^6^ Department of Burn Ruijin Hospital School of Medicine Shanghai Jiao Tong University Shanghai 200025 China

**Keywords:** atrophic gastritis, EGF, gastric stem cell, peptide, traditional Chinese medicine

## Abstract

Chronic atrophic gastritis (AG) is initiated mainly by *Helicobacter pylori* infection, which may progress to stomach cancer following the Correa's cascade. The current treatment regimen is *H. pylori* eradication, yet evidence is lacking that this treatment is effective on later stages of AG especially gastric gland atrophy. Here, using AG mouse model, patient samples, gastric organoids, and lineage tracing, this study unraveled gastric stem cell (GSC) defect as a crucial pathogenic factor in AG in mouse and human. Moreover, a natural peptide is isolated from a traditional Chinese medicine that activated GSCs to regenerate gastric epithelia in experimental AG models and revitalized the atrophic gastric organoids derived from patients. It is further shown that the peptide exerts its functions by stabilizing the EGF‐EGFR complex and specifically activating the downstream ERK and Stat1 signaling. Overall, these findings advance the understanding of AG pathogenesis and open a new avenue for AG treatment.

## Graphical abstract

This work identifies a natural peptide from a traditional Chinese medicine that activates gastric stem cells to regenerate gastric epithelia in experimental atrophic gastritis models and revitalizes the atrophic gastric organoids derived from patients. The peptide exerts its functions by stabilizing the EGF‐EGFR complex and specifically activating the downstream ERK and Stat1 signaling.

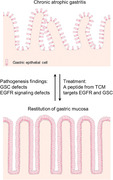

## 1 Introduction

Chronic atrophic gastritis (hereafter referred to as AG) affects up to 20% of the population.^[^ ^1^ ^]^ In addition to causing food maldigestion and iron, folate, or vitamin B12 deficiency,^[^ ^2^ ^]^ AG also represents a precursor lesion for gastric cancer, which ranks in the top five in cancer incidence and mortality worldwide.^[^ ^3^ ^]^ The common AG risk factor is *Helicobacter pylori* (*H. pylori*) infection, which induces inflammation in the antrum and parts of the gastric body and causes DNA damage in epithelial cells.^[^ ^4^ ^]^ Inflammation initially stimulates gastrin synthesis by G cells and gastric acid production by parietal cells, which, together with inflammatory cytokines, eventually leads to destruction of the gastric mucosa, especially the acid‐secreting parietal cells.^[^ ^5^ ^]^ This generates a hypochlorhydria environment,^[^ ^1^, ^2^ ^]^ favoring reproduction of *H. pylori* and other bacterial strains that promote intestinal metaplasia and gastric cancer development, as described in the Correa cascade that comprises the chronic gastritis, atrophic gastritis, intestinal metaplasia, and dysplasia/gastric carcinoma stages.^[^ ^6^ ^]^

AG patients are usually treated with antibiotics and proton pump inhibitors to eradicate *H. pylori*.^[^ ^1^, ^2^ Although the treatment downgrades AG, recent studies have shown that *H. pylori* eradication may not prevent progression from the atrophic gastritis stage to gastric cancer.^[^ ^7^ ^]^ Moreover, proton pump inhibitors may even increase the gastric cancer risk in patients with late stages of AG.^[^ ^8^ ^]^ Thus, new drugs are needed for AG treatment, especially patients at later stages of AG. This requires an in‐depth understanding of the pathogenesis of AG.

The gastric mucosa also contains chief cells, pit mucous cells, and gland mucous cells besides parietal cells and G cells, which are all derived from gastric stem cells (GSCs), under the control of niche molecules such as R‐Spondin, EGF, and BMPs.^[^ ^9^ ^]^ In the corpus, while isthmus GSCs are marked by Bmi1 or Lrig1, base‐located GSCs are marked by Troy or Lgr5.^[^ ^10^ ^]^ In the antrum, Lrig1 marks GSCs in the isthmus, while Lgr5, Sox2, Axin2, Cck2r, and AQP5 mark several GSC subpopulations at the base.^[^ ^11^ ^]^ Although *H. pylori* infection causes inflammation and DNA damage,^[^ ^1a^ ^]^ multiple studies have shown that *H. pylori* induces GSC expansion via its virulent factors^[^ ^12^ ^]^ or through induction of R‐Spondin and other niche molecules,^[^ ^11^, ^13^ ^]^ which cannot explain the loss of gastric glands in AG patients.

Herein, by analyzing AG model mice and patient samples with genetic tracing, organoid cultures, and RNA‐seq, we uncovered GSC defects as a critical AG pathogenic factor, associated with decreased signaling of EGF, an important GSC niche molecule. Testing of several traditional Chinese medicines reveals that a peptide mix enriched from Chinese traditional medicine Kangfuxin (referred to as PEEPA) contains a bioactive peptide with the potential to treat AG by revitalizing GSCs and suppressing inflammation. Kangfuxin is an ethanol extract of *Periplaneta americana* (EEPA) prescribed for repairing gastric ulcers and skin wounds in China.^[^ ^14^ ^]^ We further show that the peptide promotes gastric organoid growth and mucosa restitution via stabilizing the EGF‐EGFR complex and activating EGFR downstream ERK and Stat1, but not Akt1 or Stat3, in a slow but long‐lasting manner, compared to EGF. In addition, previous studies has shown that EGF promotes secretion of defensin 3,^[^ ^15^ ^]^ which may also contribute to the effect of Kangfuxin on AG. Moreover, we show that the peptide is stable in the acidic environment of the stomach, unlike EGF. Overall, this study advances our understanding of AG pathogenesis and identify a natural peptide with the potential to treat AG.

## 2 Results

### 2.1 PEEPA Shows Therapeutic Effects on Atrophic Gastritis in Mice

**Figure 1:**
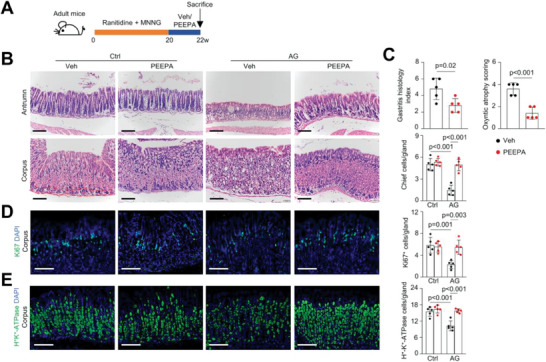
PEEPA shows therapeutic effects on atrophic gastritis model mice. A) Schedule for induction of AG in mice and treatment with PEEPA. Adult male mice were administered ranitidine (150 mg kg^−1^), exposed to drinking water containing MNNG (100 mg mL^−1^) for 20 weeks and then treated with vehicle (Veh) or PEEPA (400 µL day^−1^ per mouse) for 2 more weeks. B) H&E staining showed that PEEPA improved the gland structure in AG mouse models. C) PEEPA rescued the gastritis histology index and oxyntic atrophy score based on scores of epithelial defects, inflammation, and oxyntic cell atrophy. Data are the mean ± SD, *n* = 5 per group. D) Immunostaining revealed that PEEPA rescued the decrease in the number of proliferating cells in the corpus of AG model mice. Right panels: quantitative data. Data are the mean ± SEM, *n* = 5 per group. E) Immunostaining revealed that PEEPA rescued the decrease in the number of parietal cells. Right panels: quantitative data. Data are the mean ± SEM, *n* = 5 per group. Unpaired two‐tailed Student's *t*‐test was applied for (C), and two‐way ANOVA was applied for (C), (D), and (E). *p* < 0.05 was considered statistically significant. Scale bars: 100 µm.

Several traditional Chinese medicines, including Moludan, Sijunzi decoction, berberine have been reported to have therapeutic effects on AG while Kangfuxin (an ethanol extract of *Periplaneta americana*) shows some effects on peptic ulcers.^[^ ^16^ ^]^ We wanted to test their therapeutic effect on AG model mice generated with n‐methyl‐n‐nitro‐n‐nitrosoguanidine (MNNG) and ranitidine (**Figure** **1**A). The AG model showed loss of gastric glands, especially in the antrum, and disrupted histologic structure in the corpus, associated with increases in the gastritis histology index and oxyntic atrophic score, accompanied by a decrease in the number of proliferating epithelial cells and infiltration of immune cells (Figure 1B–D; Figure S1A,B, Supporting Information). AG model mice also showed decreases in the numbers of parietal cells, chief cells, and pit cells per gland in the corpus and decreases in Muc5ac^+^ pit cells, TFF2^+^/Muc6^+^ gland mucous cells, gastrin^+^ endocrine cells, and SST^+^ endocrine cells per gland in the antrum, as demonstrated by immunostaining and/or quantitative PCR (qPCR) analysis of lineage‐specific markers (Figure 1E; Figure S1B,C, Supporting Information). Thus, the AG mouse model displays a decrease in various epithelial cell types to similar extents.

We used Moludan, Sijunzi decoction, berberine, or the peptide mix enriched from Kangfuxin (referred to as PEEPA) to treat AG model mice and found that only PEEPA restored the gastric gland structure and diminished the gastritis histology index and oxyntic atrophic score without affecting the body weight or the histology of mouse liver and kidney (Figure 1B, C; Figure S1A,B,D, and data not shown, Supporting Information). PEEPA restored the number and integrity of the gastric glands and the number of proliferating cells, and suppressed immune cell infiltration, which appeared to be modest in the AG model (Figure 1B–D; Figure S1B, Supporting Information). PEEPA also rescued the defects in various cell types in the corpus and antrum (Figure 1E; Figure S1B,C, Supporting Information), suggesting that PEEPA alleviates AG by promoting epithelial proliferation and differentiation. However, PEEPA showed little effect on gastric glands in normal mice and other organs tested in AG model mice (Figure 1B; Figure S1B,D, Supporting Information).

### 2.2 PEEPA Promotes the Self‐Renewal of Lgr5^+^ or Bmi1^+^ GSCs in AG Models

**Figure 2:**
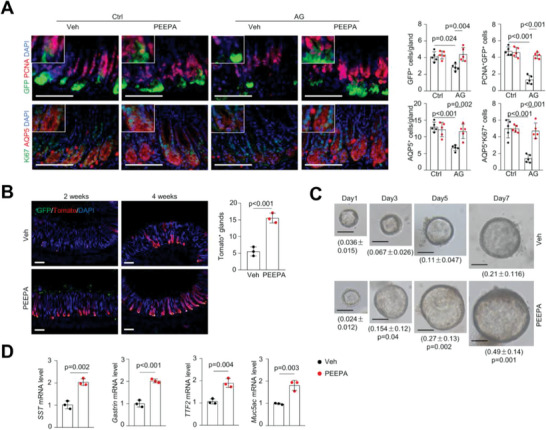
PEEPA promotes Lgr5^+^ GSC renewal and gastric organoid growth. A) Immunostaining results showed that PEEPA rescued the decrease in the numbers of Lgr5^+^ (GFP^+^), AQP5^+^, GFP^+^PCNA^+^, and AQP5^+^Ki67^+^ cells in AG models. Right panels: quantitative data. Data are the mean ± SEM, *n* = 5 per group. B) Tracing Tomato^+^ cells in *Lgr5‐GFP‐CreERT; tdTomato* AG mouse model. AG mice were treated with Veh or PEEPA for 2 and 4 weeks after 3 doses of TAM (10 mg mL^−1^). Right panel: quantitative data. Data are the mean ± SEM, *n* = 3 per group. C) A time‐course study of the effects of PEEPA on the growth of gastric organoids. Quantitative data are labelled below. Four wells were counted for each sample. Data are the mean ± SD, *n* = 3 per group. D) qPCR results revealed that PEEPA promoted the expression of lineage‐specific genes in organoid cultures. Data are the mean ± SD, *n* = 3 per group. Unpaired two‐tailed Student's *t*‐ test was applied for (B), (C), and (D), and two‐way ANOVA was applied for (A). *p* < 0.05 was considered statistically significant. Scale bars: 100 µm.

The decrease in various epithelial cell types in AG model mice suggests defects in GSCs, which maintain the gastric glands.^[^ ^10^, ^11^, ^12^ We used *Lgr5‐GFP‐CreERT* mice, which have antral GSCs marked by GFP, to induce AG. We found that the number of GFP^+^ GSCs was substantially reduced in the antrum of AG models, as were proliferating Lgr5^+^ cells (**Figure** **2**A). Immunostaining also revealed a decrease in the numbers of AQP5^+^ GSCs, which partially overlapped with Lgr5^+^ GSCs,^[^ ^11e^ ^]^ and proliferating AQP5^+^ cells in AG model mice (Figure 2A). These results suggest that atrophic gastritis is associated with impairment of GSC self‐renewal in the antrum.

In PEEPA‐treated *Lgr5‐GFP‐CreERT* AG model mice, the numbers of GFP^+^ cells and proliferating GFP^+^ cells were restored to close to normal levels, as were the numbers of AQP5^+^ cells and proliferating AQP5^+^ cells (Figure 2A). Notably, PEEPA showed little effect on GSCs in normal mice (Figure 2A). Moreover, a genetic tracing experiment using *Lgr5‐GFP‐CreERT;Rosa‐Tomato* AG model mice (4 weeks after tamoxifen (TAM) administration) revealed that PEEPA increased Lgr5^+^ GSC‐mediated renewal, manifested by an increase in the number of Tomato^+^ GSC daughter cells in the gastric glands (Figure 2B).

We also generated an AG model in *Bmi1‐CreERT;tdTomato* mice, which marked corpus GSCs but not antral GSCs. Our tracing experiments revealed that PEEPA increased the activity of Bmi1^+^ GSCs, as manifested by an increase in the number of Tomato^+^ cells in the corpus (Figure S1E, Supporting Information). Thus, PEEPA activated several GSC populations in both the antrum and corpus and improved their renewal activities in AG model mice, suggesting that GSCs can be activated to restore the atrophic gastric glands. The proportional increase of various cell types suggests that PEEPA mainly acts on GSCs rather than differentiated cells.

### 2.3 PEEPA Promotes Gastric Organoid Growth

To validate the direct effects of PEEPA on GSCs, we used gastric organoids as a model, which are widely used to assess the activities of GSCs.^[^ ^17^ ^]^ We isolated epithelial cells, including GSCs, from the antrum of normal mice and cultured them in DMEM/F12 advanced medium containing R‐spondin1, EGF, noggin, FGF10, and Wnt3a.^[^ ^11^, ^17^ ^]^ We added different concentrations of PEEPA and found that PEEPA was toxic at a 1:20 dilution, yet it increased the size of the organoids at a 1:100 dilution (Figure 2C; Figure S2A, Supporting Information). qPCR revealed that the expression of lineage‐specific marker genes, including *SST* (encodes somatostatin), *gastrin*, *TTF2*, and *Muc5ac*, was significantly increased (Figure 2D). Another batch of PEEPA showed similar effects on organoid growth (Figure S2B,C, Supporting Information). These results suggest that PEEPA directly promotes GSC self‐renewal and that PEEPA mainly acts on GSCs rather than differentiated cells.

### 2.4 RNA‐Seq Analysis Reveals that PEEPA Activates Mitogenic Signaling

**Figure 3:**
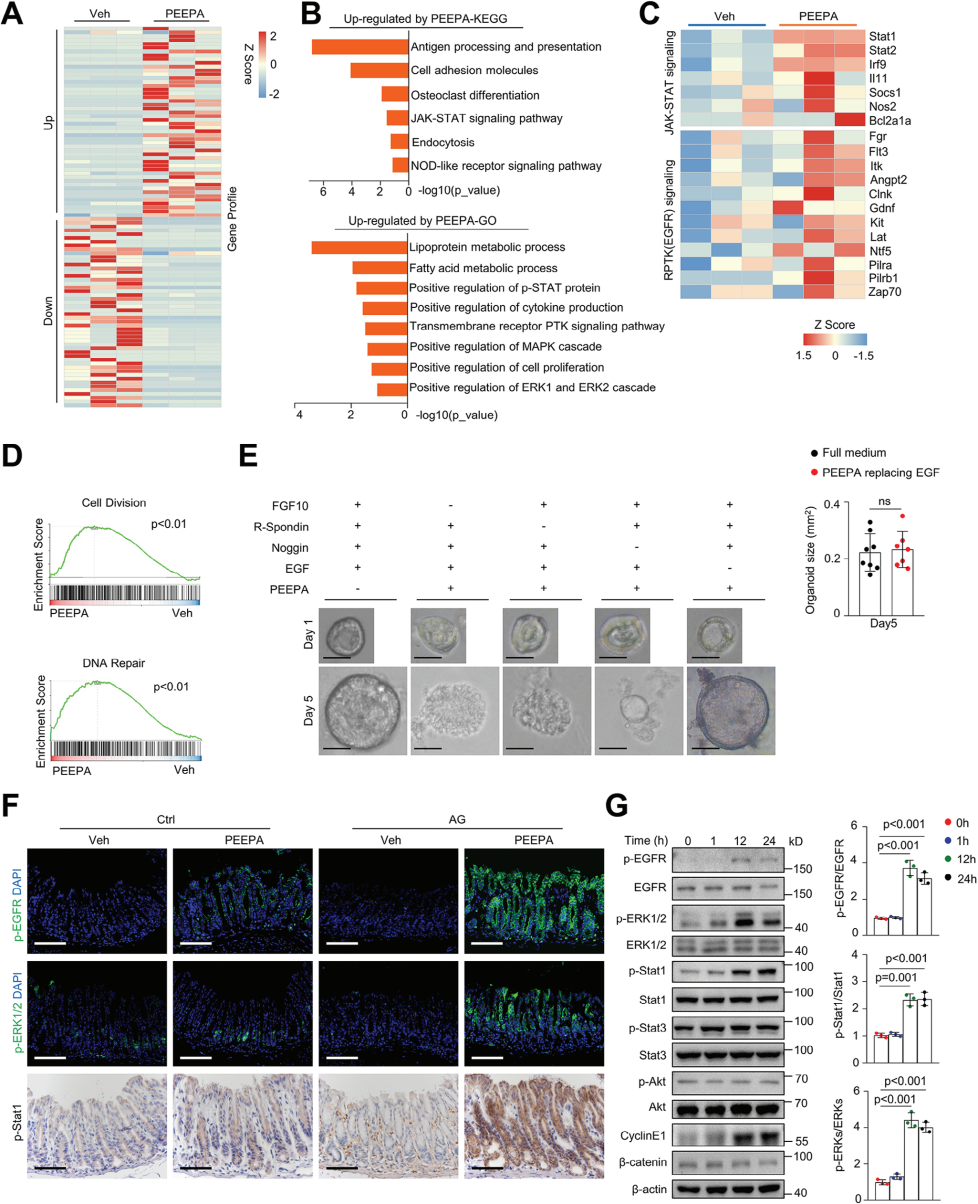
PEEPA activates EGF signaling pathways with unique dynamics. A) The transcriptomes of gastric epithelial cells were altered by PEEPA. Three AG model mice were treated with PEEPA or Veh via oral gavage. B) KEGG and GO analyses showed enhanced signaling pathways and modules in the PEEPA‐treated group. C) Heatmap results showed alterations in the expression of receptor protein tyrosine kinase signaling and Jak‐Stat signaling pathway genes induced by PEEPA. D) The GSEA results showed that cell division‐ and DNA repair‐related genes were upregulated by PEEPA. E) Organoid culture results showed that PEEPA substituted for EGF but no other factors. Right panel: quantitative data. Data are the mean ± SD, *n* = 3 per group. F) Representative immunostaining data showing p‐EGFR, p‐Stat1, and p‐ERK1/2 in stomach sections of PEEPA‐treated normal or AG model mice. G) Western blot data revealed that PEEPA activated EGFR, ERK1/2 and Stat1 but not Stat3 in GES‐1 cells. Right panel: quantitative data. Data are the mean ± SD, *n* = 3 per group. Unpaired two‐tailed Student's *t*‐test was applied for (F) and (G), and *p* < 0.05 was considered statistically significant. Scale bars: 100 µm.

To understand how PEEPA functions in AG model mice, we performed bulk RNA sequencing (RNA‐seq) on gastric samples of AG model mice treated with PEEPA for three days to focus on the primary effects of PEEPA. PEEPA altered the expression of a few hundred genes (**Figure** **3**A). KEGG and GO analyses revealed that cell proliferation, receptor tyrosine kinases especially EGFR, the Jak‐Stat pathway, the NOD‐like pathway, antigen presentation, and the ERK pathway were activated, whereas the Ca2^+^ and PKG pathways, amino acid metabolism, immune response, and muscle contraction‐related genes were suppressed (Figure 3B,C; Figure S3A–C, Supporting Information). Note that we did not detect alteration in signaling pathways of Wnts or BMPs, which are important GSC niche molecules. Many cytokines and chemokines were downregulated, consistent with the observation that PEEPA alleviated immune cell infiltration in AG model mice (Figure S3D, Supporting Information). In addition, gene set enrichment analysis (GSEA) confirmed that cell division‐ and DNA repair‐related genes were enriched by PEEPA (Figure 3D). Thus, PEEPA may activate mitogenic pathways, including EGFR, Jak‐Stat, and ERK, to promote cell proliferation. The observation that no epithelial differentiation program was enriched in the transcriptome analysis supports that PPEPA may not directly target epithelial differentiation.

### 2.5 PEEPA Acts as EGF in Organoid Cultures and Activates EGFR Signaling

We then tested whether PEEPA could replace EGF, Wnt3a, FGF10, or noggin in gastric organoid cultures. We found that PEEPA could only substitute for EGF (Figure 3E), suggesting that PEEPA contains bioactive ingredients with activities of EGF, a GSC niche molecule.^[^ ^10c^ ^]^ Indeed, EGF but not FGF, noggin, or Wnt is known to activate both the MAPK and Jak‐Stat pathways.^[^ ^18^ ^]^ To verify this finding, we analyzed the stomach sections and protein samples of PEEPA‐treated AG and control mice and found that EGFR, ERK, and Stat1, but not Stat3 or Akt1, were activated by PEEPA (Figure 3F; Figure S3E, Supporting Information). Stat1 has modest pro‐proliferative and immunomodulatory activities compared to Stat3.^[^ ^19^ ^]^ We also used the gastric epithelial cell line GES‐1 to verify these findings. Western blot analysis revealed that PEEPA (different batches) activated EGFR, ERKs, and Stat1 but not Akt1 or Stat3 (Figure 3G; Figure S3F, Supporting Information). Overall, these results suggest that PEEPA acts as EGF, although activation of the EGFR downstream signaling profile is not identical.

### 2.6 PEEPA‐Induced Organoid Growth Requires Jak‐Stat and ERK Signaling

The above results indicate that PEEPA activates ERKs and Stat1, although with much slower dynamics (Figure 3G), both of which are pro‐mitogenic.^[^ ^18^, ^20^ ^]^ To answer the question whether these two pathways contributed to PEEPA‐induced organoid growth, we first tested whether combined ERK1 and Stat1 agonists^[^ ^21^ ^]^ could substitute EGF in organoid cultures and found that these agonists promoted organoid growth in the absence of EGF (Figure S4A, Supporting Information). We then inhibited Jak‐Stat1 signaling with the Jak inhibitor ruxolitinib (Ruxo). We found that Ruxo impeded PEEPA‐induced organoid growth and differentiation (Figure S4B,C, Supporting Information). The ERK inhibitor U0126 also suppressed PEEPA‐induced organoid growth (Figure S4D, Supporting Information). In addition, we found that the EGFR inhibitor afatinib blocked the pro‐growth effect of PEEPA on gastric organoids (Figure S4E, Supporting Information). These results, together with the observation that the inhibitors showed less effect on the growth of organoids without PEEPA, suggest that the highly activated EGFR‐Stat1 and EGFR‐ERK pathways mediate the pro‐proliferative activity of PEEPA.

### 2.7 Identification of EGFR‐Binding Peptides via Affinity‐Mass Spectrometry

**Figure 4:**
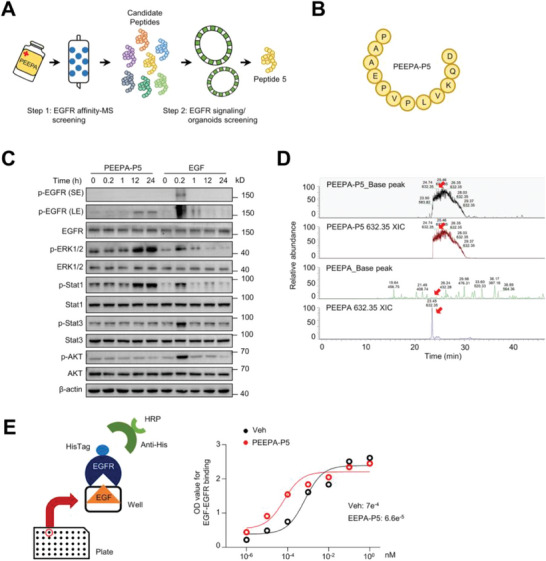
Identification of EGFR‐binding peptides. A) A diagram showing the strategy to identify EGFR‐binding peptides in PEEPA. B) The sequence of P5 identified by immune‐affinity chromatography‐tandem mass spectrometry. C) Western blot results showed activation of the EGFR‐Stat1 axis by PEEPA‐P5 (0.4 µm) in comparison to EGF (0.4 µm) in GES‐1 cells. D) Mass spectrometry analysis detected P5 in PEEPA. PEEPA was dried and dissolved in 0.1% TFA, and the saline was removed by ZipTip‐C18. The samples were redissolved in 0.1% FA/2% CAN and analysed on an Orbitrap Fusion LUMOS mass spectrometer. Synthesized P5 was used as a standard. Red arrows represent the rate of m/z (632.35). E) ELISAs showed that PEEPA‐P5 promoted the binding of EGF and EGFR. EGF proteins were coated on the plates, and EGFR containing a His‐tag was added, together with different concentrations of PEEPA‐P5 or Veh. The OD value of each sample was measured by horseradish peroxidase. Data are the mean ± SD, *n* = 3 per group, *p* value = 0.027.

To identify the bioactive components of PEEPA, we used an affinity capture‐mass spectrometry approach with the extracellular domain of EGFR as bait (**Figure** **4**A). We identified 7 peptides from PEEPA (designated PEEPA‐P1 to P7) (Figure 4B; Figure S5A, Supporting Information). We synthesized these peptides and tested their activities on GES‐1 cells using p‐Stat1 as an indicator. We found that PEEPA‐P1 and P5 could activate Jak‐Stat1 signaling, with P5 showing the greatest effect (Figure 4C; Figure S5B,C, Supporting Information). Similar to PEEPA, P5 activated ERKs and Stat1 with slower dynamics than EGF without activating Akt1 or Stat3 (Figure 4C). We then focused on P5 in the following studies.

Mass spectrometry analysis revealed that PEEPA contained various peptides (Figure 4D). PEEPA‐P5 was detected in PEEPA (both batches), although at low levels (Figure 4D; Figure S5D, Supporting Information). Intriguingly, a search of the PubMed database failed to find a match for P5 (Table S1, Supporting Information). One explanation is that the natural peptide is encoded by noncoding RNAs. Alternatively, the *P. americana* genome may have not been exhaustively sequenced. Certainly, this issue warrants further investigation. Nonetheless, PEEPA‐P5 has no homology to EGF molecules.

### 2.8 PEEPA‐P5 Stabilizes the EGF‐EGFR Complex and Alters the Signaling Profile

We determined the affinity between PEEPA‐P5 and the extracellular domain of human EGFR using surface plasmon resonance (SPR) and found that PEEPA‐P5 had a low affinity for EGFR compared to EGF (Figure S6A, Supporting Information). Since PEEPA‐P5 activated ERK and Jak‐Stat1 signaling with slow dynamics, we speculated that PEEPA‐P5 might affect the EGF‐EGFR complex. Indeed, in vitro EGF‐EGFR interaction assays revealed that PEEPA‐P5 increased the EGF‐EGFR interaction by 10.6‐fold (Figure 4E), suggesting that PEEPA‐P5 stabilizes the EGF‐EGFR complex, which may underlie the prolonged activation of EGFR and altered spectrum of downstream signaling events. The EGF might be provided by serum in the culture medium or secreted by the gastric epithelial cells in the organoids. Serum contains low levels of various growth factors including EGF.^[^ ^22^ ^]^ We confirmed that the serum could activate EGFR in GES‐1 cells (Figure S6B, Supporting Information). Thus, PEEPA and PEEPA‐P5 may promote serum‐contained EGF‐EGFR interaction and activate the downstream signaling. Similarly, modified Parathyroid hormone‐receptor interaction has been reported to alter the signaling profile.^[^ ^23^ ^]^

We then conjugated PEEPA‐P5 to fluorescein isothiocyanate isomer (FITC) and found that the peptide was mainly detected at the cell surface in cultured cells (Figure S6C, Supporting Information), consistent with its interaction with the EGF‐EGFR complex. Using P5‐fluorescein, we found that PEEPA‐P5 had a retention time of 3.09 h in the stomach (Figure S6D, Supporting Information), which also suggests that PEEPA‐P5 is relatively stable in the acidic environment of the stomach. Moreover, we found that the half‐maximal inhibitory concentration (IC50) for PEEPA‐P5 on GES‐1 cells was 284.4 µM (Figure S6E, Supporting Information), suggesting that it has low toxicity.

### 2.9 PEEPA‐P5 has PEEPA‐Like Activities in Organoid and AG Model Mice

**Figure 5:**
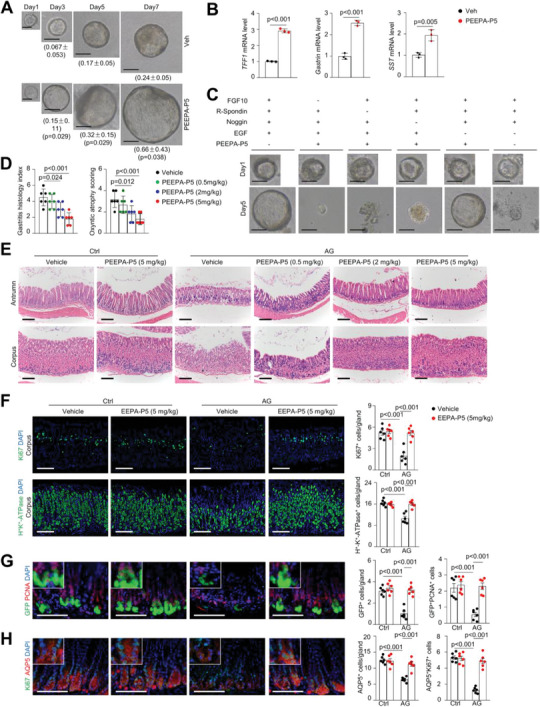
PEEPA‐P5 has similar activities as PEEPA in gastric organoids and AG mouse models. A) Consecutive images showed the effects of PEEPA‐P5 on the growth of gastric organoids. Quantitative data are shown below. Data are the mean ± SD, *n* = 3 per group. B) qPCR results showed the effects of PEEPA‐P5 on cell differentiation in gastric organoids. Data are the mean ± SD, *n* = 3 per group. C) PEEPA‐P5 could substitute for EGF in organoid culture. D) PEEPA‐P5 rescued the gastritis histology index and oxyntic atrophy score. Data are the mean ± SD, *n* = 6 per group. E) Histologic analysis results showed that PEEPA‐P5 effectively rescued the structural defects in the antrum and corpus of AG models. Mice were administered Veh or PEEPA‐P5 (0.5, 2, and 5 mg kg^−1^) in the AG mouse model. F) Representative immunostaining results showed that PEEPA‐P5 rescued the decrease in the number of proliferating cells and parietal cells in AG model mice. Right panels: quantitative data. Data are the mean ± SEM, *n* = 6 per group. G) Immunostaining results showed that PEEPA‐P5 rescued the decrease in the numbers of Lgr5^+^ cells and proliferating Lgr5^+^ cells. Right panel: quantitative data. Data are the mean ± SEM, *n* = 6 per group. H. Immunostaining results showed that PEEPA‐P5 rescued the decreases in the numbers of AQP5^+^ GSCs and proliferating AQP5^+^ cells. Right panel: quantitative data. Data are the mean ± SEM, *n* = 6 per group. Unpaired two‐tailed Student's *t*‐test was applied for (A), (B), and (D), and two‐way ANOVA was applied for (F), (G), and (H). *p* < 0.05 was considered statistically significant. Scale bars: 100 µm.

PEEPA‐P5 also increased organoid growth and promoted the differentiation of various cell types in the organoids, similar to PEEPA (**Figure** **5**A,B). Importantly, PEEPA‐P5 was able to substitute for EGF in organoid cultures (Figure 5C). Moreover, this peptide restored the number and size of gastric glands and reduced the gastritis histology index and oxyntic atrophic score in a dose‐dependent manner in mice with AG (Figure 5D,E), although it showed no obvious effect on the liver or kidney (Figure S7A,B, Supporting Information). PEEPA‐P5 also rescued the numbers of proliferating cells and various cell types in mice with AG as well as the activation of EGFR, Stat1, and ERKs, although it showed little effect on normal mice (Figure 5F; Figure S8A,B, Supporting Information). In addition, PEEPA‐P5 suppressed immune cell infiltration (Figure S8A, Supporting Information). Importantly, the numbers of Lgr5^+^ GSCs and AQP5^+^ GSCs were restored by PEEPA‐P5 in AG model mice, as were the numbers of proliferating Lgr5^+^ cells and AQP5^+^ cells (Figure 5G,H). These results validated its positive effects on GSC self‐renewal.

We also carried out RNA‐seq on gastric samples of the P5‐treated mice with AG and found that P5 enhanced the gene modules/pathways including cell proliferation and division, EGFR signaling, MAPK signaling, and Jak‐Stat signaling and suppressed the expression of genes controlling amino acid metabolism, leukocyte migration and adhesion, and response to cytokines (Figure S9A–E, Supporting Information), many of which overlapped with those of the PEEPA‐treated samples, confirming that P5 has similar activities as PEEPA.

### 2.10 AG Patient Samples Show Defects in Gastric Stem Cells and EGF Signaling

**Figure 6:**
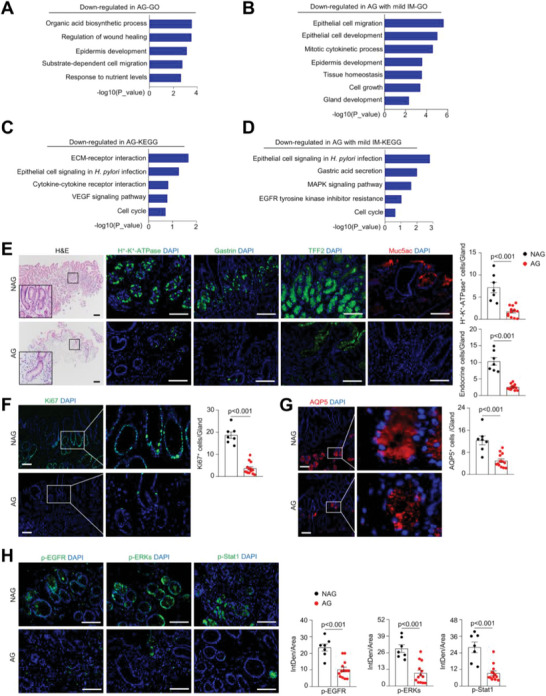
Human AG samples showed defects in GSCs and EGFR‐Stat1 signaling. A,B) GO analysis revealed the decreased modules in human AG (A) and AG with IM (B) compared to NAG based on scRNA‐seq data. Data from 3 AG biopsies, 2 AG biopsies with IM, and 3 NAG biopsies were used (GEO: GSE134520). C,D) KEGG pathway analysis revealed downregulated signaling pathway genes in human AG (C) and AG with IM (D) compared to NAG. E. AG patient samples showed a decline in the thickness of the gastric mucosa and the number of H^+^‐K^+^‐ATPase‐expressing parietal cells, gastrin^+^ endocrine cells, TFF2^+^ mucous cells, and Muc5ac^+^ pit cells. Right panels: quantitative data. *n* = 7 for NAG and *n* = 13 for AG. F) AG patient samples showed a decline in the number of proliferating cells. Right panels: quantitative data. *n* = 7 for NAG and *n* = 13 for AG. G) Representative immunostaining results showed that AG patient samples displayed a decrease in the number of AQP5‐expressing GSCs. Right panels: quantitative data. *n* = 7 for NAG and *n* = 13 for AG. H) Analysis of EGFR, ERK1/2, and Stat1 activation in AG patient samples. Right panels: quantitative data. IntDen means integrated density. *n* = 7 for NAG and *n* = 13 for AG. Unpaired two‐tailed Student's *t*‐test was applied for (E), (F), (G), and (H), and *p* < 0.05 was considered statistically significant. Scale bars: 100 µm.

We then wanted to test whether human AG had similar defects in GSCs. Several studies have compared biopsied AG patient samples with nonatrophic gastritis (NAG) samples as controls at the single‐cell level.^[^ ^24^ ^]^ We re‐examined the clustering of gastric cells and found a decrease in the percentages of various cell types, including CCK2R^+^ stem cells, in AG and AG with mild intestinal metaplasia (IM) samples (Figure S10A–C, Supporting Information). Our GO analysis revealed that AG epithelial cells showed enriched expression of genes in the immune response pathways, citric acid cycle, and fatty acid metabolism, and decreased modules included wound healing, epithelial cell proliferation, MAPK signaling, EGFR signaling, and gland development but not Wnt or BMPs signaling (**Figure** **6**A,B; Figure S10D,E, Supporting Information). KEGG analysis showed enhanced inflammatory signaling and decreased pathways including gastric acid secretion, cell cycle, and the MAPK pathway, in AG cells (Figure 6C,D; Figure S10F,G, Supporting Information). These results suggest that human AG is also associated with defects in GSCs and pro‐mitogenic signaling.

To verify the above findings, we collected 38 patient antrum samples (13 AG and 7 NAG samples for histologic analysis; 12 AG and 6 NAG samples for organoid culture) (Table S2, Supporting Information). H&E and immunostaining for H^+^‐K^+^‐ATPase or Ki67 revealed decreases in the size and number of the gastric glands in the antrum and the numbers of proliferating cells and parietal cells; moreover, the numbers of pit cells, gland mucous cells, and cells positive for AQP5, which marks human GSCs,^[^ ^11e^ ^]^ were reduced compared to those of the control samples (Figure 6E–G). These results confirmed the existence of GSC defects in AG patient samples. Thus, both AG mouse model and patient sample studies suggest that GSC self‐renewal defects constitute a critical element in AG pathogenesis.

We also stained the patient antrum sections for p‐EGFR, p‐ERK, and p‐Stat1 and found that activation of these signaling molecules was reduced in AG samples (Figure 6H), suggesting that the EGFR‐Stat1/ERK pathway may be involved in the pathogenesis of AG in humans.

### 2.11 PEEPA‐P5 Promotes the Growth of Gastric Organoids Derived from AG Patients

**Figure 7:**
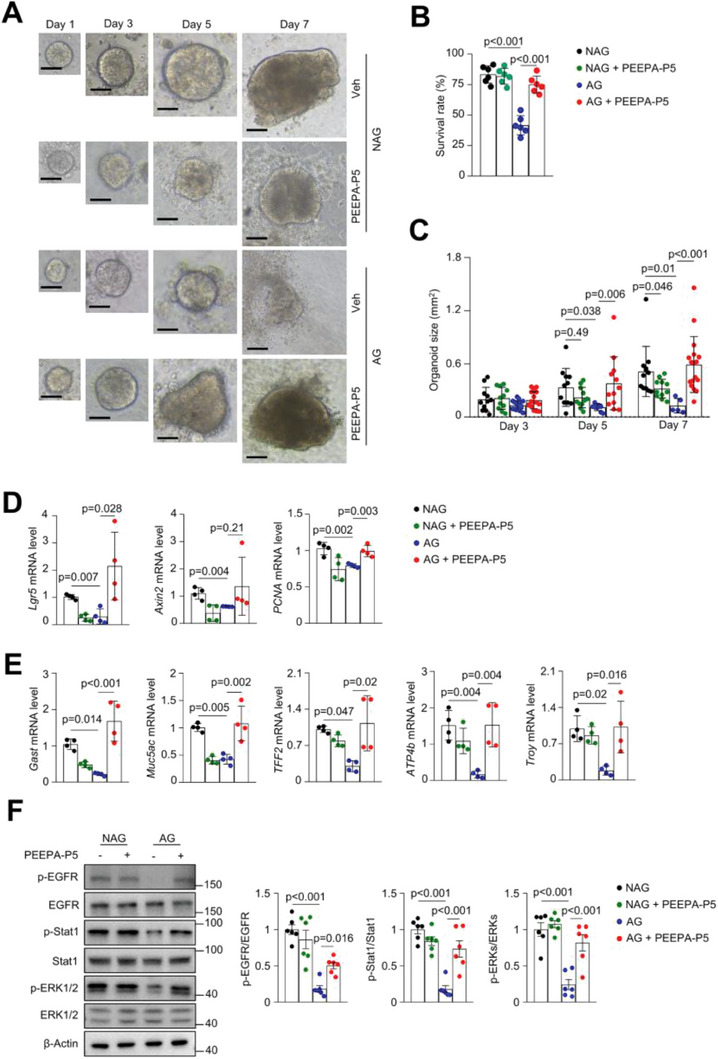
PEEPA‐P5 specifically promotes the growth of gastric organoids derived from AG patients. A) Representative images show the effects of PEEPA‐P5 on the growth of patient‐derived gastric organoids. B) The survival rate of gastric organoids derived from patients. Data are the mean ± SD, *n* = 6 for NAG and *n* = 12 for AG. C) Quantitative data of organoid size (A). Data are the mean ± SD, *n* = 6 for NAG and *n* = 12 for AG. D) qPCR results revealed that PEEPA‐P5 rescued the expression of *Lgr5* and *Pcna* in gastric organoids derived from AG patients. Data are the mean ± SD, *n* = 4 per group. E) qPCR results revealed that PEEPA‐P5 rescued the expression of differentiation marker genes in gastric organoids derived from AG patients. Data are the mean ± SD, *n* = 4 per group. F) Western blot results showed that EGFR‐Stat1 and EGFR‐ERK signaling was activated by PEEPA‐P5 (0.4 µm) in AG patient‐derived gastric organoids but not in NAG organoids. Data are the mean ± SD, *n* = 6 per group. Two‐way ANOVA was applied for (C), (D), (E), and (F). *p* < 0.05 was considered statistically significant. Scale bars: 100 µm.

To test the possible effects of PEEPA‐P5 on human AG, we took advantage of gastric organoid cultures. We collected biopsied antrum samples, isolated epithelial cells, and cultured them in Matrigel containing various growth factors. While the organoids derived from NAG samples survived and expanded, gastric organoids from a large portion of AG patients failed to survive, and the rest showed a reduction in growth and expression of differentiation marker genes (**Figure** **7**A–C), indicating that GSCs have largely lost renewal activity in AG patients.

PEEPA‐P5 significantly increased the survival rate and growth of the organoids derived from AG patients, although it slightly inhibited the growth of organoids derived from NAG patients (Figure 7A–C), supporting that the pathogenesis of AG differs from that of NAG. qPCR analysis revealed that organoids from AG patients showed defects in the expression of the proliferation‐related gene *Pcna* and GSC marker *Lgr5* and *Axin2* (Figure 7D). Notably, PEEPA‐P5 suppressed the expression of *Lgr5* and *Axin2* in NAG samples, consistent with its negative effect on the growth of organoids derived from NAG patients (Figure 7A,D). Since there exists crosstalk between EGF signaling and Wnt signaling,^[^ ^25^ ^]^ prolonged EGFR activation by P5 may eventually have let to suppressed Wnt signaling and reduced expression of *Lgr5* and *Axin2*, target genes of Wnt signaling. This certainly needs further investigation. Moreover, organoids from AG patients showed decreased expression of differentiation marker genes of parietal, pit, and chief cells, which were partially rescued by PEEPA‐P5 treatment (Figure 7E). These results suggest that PEEPA‐P5 specifically promotes the proliferation and differentiation of GSCs from AG patients but not those from NAG patients.

What caused the differential responses in organoids from AG and NAG patients? Our scRNA analysis and immunostaining results showed that EGFR downstream signaling molecules including p‐EGFR, p‐Stat1, and p‐ERK, were suppressed in the samples from AG patients compared to NAG patients (Figure 6A–D,H). We tested the activation of EGFR signaling in response to P5 in organoid cultures and found that organoids from AG patients showed severely suppressed EGFR signaling, which could be boosted by P5 (Figure 7F). On the other hand, the organoids from NAG patients showed much stronger activation of the EGFR signaling under normal culture conditions, which could not be enhanced by P5 (Figure 7F). These results suggest that the organoids from AG patients have lost the sensitivity to EGF, due to unknown defects in the GSCs, yet they respond to P5, which has a unique mechanism of action on EGFR signaling. The differences in the sensitivity to P5 explain why only AG organoids responded to P5, which certainly warrants further investigation.

## 3 Discussion

AG is a prevalent disease that not only impairs food digestion but also increases the risk for gastric cancer development. The current treatment regimens, which mainly target *H. pylori*, may not help in restoring the mucosal structure and preventing progression to gastric cancer in AG patients who are already at atrophic or intestinal metaplasia stage.^[^ ^7^ ^]^ Thus, identification of new drugs for AG treatment is urgently needed. Our current study suggests that AG pathogenesis involves GSC self‐renewal defects. Moreover, we identified a natural peptide from traditional Chinese medicine Kangfuxin that shows therapeutic effects on experimental AG and gastric organoids derived from AG patients by rebooting GSCs. This study not only identifies a peptide that has therapeutic potential on AG but also deepens our understanding of AG aetiology.

We also show that the numbers of all cell types per gland are decreased in AG mouse models, AG patient samples, and gastric organoids derived from AG patients and that these phenotypes are associated with self‐renewal defects in GSCs, including GSCs marked by Lgr5, Bmi1, or AQP5 in mice and CCK2R^+^ or AQP5^+^ GSCs in AG patient samples. The proportional increase of various cell types suggests that PEEPA mainly acts on GSCs rather than differentiated cells. This is in contrast to the general belief that the G cells and parietal cells are the main cell types destructed in AG,^[^ ^5a–c^ ^]^ thus deepening our understanding of AG pathogenesis. Although our results appear to contradict the observation that *H. pylori* induces GSC expansion,^[^ ^12^ ^]^ the loss of GSCs may be a long‐term consequence of *H. pylori* infection, while GSC activation by *H. pylori* is an acute response. Our findings suggest for the first time that GSCs play important roles in AG aetiology.

What is the molecular basis of GSC self‐renewal defects in AG? Our studies show that GSC defects are associated with suppression of the mitogenic EGFR‐ERK and EGFR‐Stat1 pathways but not BMP‐Smad or Wnt signaling, consistent with studies showing a reduction in EGFs in AG samples^[^ ^5^, ^26^ ^]^ but not a study reporting the opposite results.^[^ ^27^ ^]^ The discrepancy may be caused by the use of AG samples of different disease stages,^[^ ^15b^ ^]^ or analysis of single members of the EGF family. In this study, we analyzed the downstream signaling pathways of EGF receptor, which represent the combined effect of EGF family members, as well as feedback regulations and crosstalk from other signaling pathways. Our scRNA analysis, immunostaining, and organoid cultures showed that EGFR downstream signaling molecules including p‐EGFR, p‐Stat1, and p‐ERK, were suppressed in AG patients compared to NAG patients (Figure 6A–D,H and Figure 7F). Moreover, we show that activation of EGFR signaling by the PEEPA peptide revitalizes GSCs and restitutes the gastric mucosa. In addition, we show that both PEEPA and the peptide suppressed immune cell infiltration in AG model mice, consistent with the observations that EGFR activation stimulates secretion of defensin molecules and modulates immune response.^[^ ^15^, ^28^ ^]^

Interestingly, the natural PEEPA peptide shows no homology to EGF molecules. Moreover, PEEPA‐P5 has unique properties compared to EGF. First, PEEPA‐P5 has low affinity for EGFR, but it enhances the EGF‐EGFR interaction; therefore, its effect relies on endogenous EGF and EGFR. Second, while EGF activates ERK, Akt1, and Stat1/3 signaling, PEEPA or PEEPA‐P5 mainly activates ERK and Stat1. Third, PEEPA‐induced ERK and Stat1 activation is much slower. These results, together with the instability of EGF in acidic stomach environment and rapid endocytosis and degradation of EGF‐EFGR complex, suggest that PEEPA‐P5 is not replaceable by EGF in AG treatment. Moreover, the observations that Stat1 has weaker pro‐proliferative activity than Stat3,^[^ ^19^ ^]^ the peptide has relatively short retention time in the stomach, and that EGFR activity is weak in lower part of gastric glands suggest that the PEEPA peptide may have low oncogenic activities.^[^ ^9a^ ^]^ We also show that PEEPA or P5 does not stimulate growth of the gastric glands in normal mice. Intriguingly, P5 stimulates the growth of gastric organoids derived from AG patients but not organoids from NAG patients, consistent with the notion that AG and NAG have different pathogeneses. Further studies show that organoids from NAG patients are highly sensitive to EGF while organoids from AG patients are insensitive due to unidentified GSC defects. However, organoids from AG patients are still capable of responding to P5, likely due to the unique action mechanism of the peptide.

In summary, our studies, based on mouse models, RNA‐seq, patient samples, and gastric organoids, reveal GSC defects as an integral component in AG pathogenesis. We, for the first time, have identified a new drug candidate that targets GSCs and restores gastric mucosal integrity to treat AG. Our findings suggest that P5 has therapeutic potentials. However, this drug may cause tumor at high dose or after prolonged use. The safety of the drug candidate thus needs careful monitoring. These findings suggest that a combination of *H. pylori* eradication and the PEEPA peptide, which targets different stages of AG, may be more effective. This study also presents an example that uses modern biological approaches to study traditional Chinese medicines.

## 4 Experimental Section

### Mouse Lines

The mouse experiments were carried out following the recommendations from the National Research Council Guide for the Care and Use of Laboratory Animals. All protocols were approved by the Institutional Animal Care and Use Committee of Shanghai Jiao Tong University, China [SYXK(SH)2011‐0112]. *Lgr5‐GFP‐CreERT* mice, *Bmi1‐CreERT* and *Rosa‐tomato* mice were purchased from the Jackson Laboratory, and C57BL/6 mice were purchased from Model Organisms, Shanghai.

### Genetic Tracing Experiments

*Lgr5‐GFP‐CreERT; tdTomato* and *Bmi1‐CreERT; tdTomato* AG mouse model were treated with Veh or PEEPA for 2 and 4 weeks after 3 doses of TAM (10 mg kg^−1^) intraperitoneal injection.

### Human Gastritis Samples

Human nonatrophic gastritis and chronic atrophic gastritis tissues were provided by the Department of Gastroenterology, Ruijin Hospital, China. Protocols were approved by the Research Ethics Committee of Ruijin Hospital. The samples were classified into nonatrophic and chronic atrophic gastritis based on the score of inflammation level, activity, atrophy, and intestinal metaplasia according to the consensus criteria for the diagnosis of gastritis. The clinicopathological parameters of all patients are summarized in Table S2 (Supporting Information). Written informed consent was obtained from all participants.

### Mouse Atrophic Gastritis Model

For establishment of a mouse model of atrophic gastritis, normal mice had free access to water containing N‐methyl‐N‐nitro‐N‐nitrosoguanidine (MNNG, 100 mg mL^−1^). Moreover, the mice were given 400 µL of ranitidine (8 mg mL^−1^) in solution at a dose of 150 mg kg^−1^ daily. After 20 weeks, the mice were euthanized and examined.

### PEEPA and Peptides Treatment

Periplaneta americana insects were cultivated in an industrialized facility and the ethanol extract of (PEEPA) was prepared with a standardized method, which was dissolved in the vehicle containing water, 15% glycerol and 0.2% potassium sorbate. PEEPA‐derived peptides (PEEPA‐P5) were dissolved to 500 ng mL^−1^ in water for in vitro experiments and to 1 mg mL^−1^ for in vivo experiments. Mice were administrated with PEEPA, PEEPA‐P5, or vehicle through oral gavage daily for 2 or 4 weeks.

### Gastric Organoid Culture

The gastric antrum of mice was harvested and the middle part was cut open. After cleaning in PBS containing 1% pencillin‐streptomycin (PS) for several times, the food residue and other impurities were completely removed. The gastric gland epithelial layer was separated from the muscle layer with forceps under the stereoscope. The epithelial layer was cut into 2.5 mm long fragments, which were digested in 2.5 mm EDTA/DPBS at 4 °C for 1 h. The gland fragments were filtered through a 70 m strainer (Thermo Fisher), washed with cold DPBS buffer, and centrifuged at 1000 rpm for 5 min. Afterward, the gland sediments were suspended in growth factor reduced phenol‐free Matrigel (Corning) and seeded into 48‐well plate. The organoids were cultured in DMEM/F12 advanced medium containing R‐Spondin1, EGF, Noggin, FGF10, and Wnt3a. PEEPA in final concentration of 1% or PEEPA‐P5 in 0.38 m was added at Day 1. At different time points (Day 1, 3, 5, and 7), the organoids were observed under inverted microscope, and the organ diameter and bud numbers were measured to determine the levels of cell proliferation. At Day 7, the organs were collected and total RNA was extracted. For human gastric organoids, biopsy samples were digested by Dispase II and Collagenase XI for 30 min. After centrifuge, gland was cultured in the above‐mentioned organoid medium extra‐supplemented Gastrin and inhibitor A83‐01. For Western blot analysis of human gastric organoids, 12 biopsy samples (6 NAG and 6 AG) were collected. Each biopsy sample was cultured in 6 independent wells after digestion (3 for veh and 3 for PEEPA‐P5 treatment). At Day 7, the organoids were collected from 3 treated wells for one sample containing 50–100 organoids and proteins were extracted for Western blot analysis.

For JAK‐Stat, MEK1 and EGFR inhibition, organoid cultures were respectively treated with Ruxotilinib (15 µm, Selleck, #s1039), U0126 (20 µm, Selleck, #s1102) and Afatinib (1 µm, Selleck, #s1011). The inhibitor was prepared following the manufacturer's instruction.

### Growth Factor Substitution Experiments

The organoids cultured described above were divided into several groups with medium containing different combination of the 5 growth factors including control group (all 5 growth factors), R‐Spondin‐replaced group, Noggin‐replaced group, EGF‐replaced group, and FGF10‐repalced group. In these groups, either PEEPA or PEEPA‐P5 was used to replace each of these factors. The cultured organoids were observed under a microscope and growth rates were determined at different time points (Day 1 and 5). For Stat1 and MEK1 agonist studies, organoid cultures were treated with 2‐NP (45 µm, MCE, #HY‐W013523) and PAF‐C16 (1 µm, MCE, #HY‐108635) to substitute EGF.

### ELISA Assay for EGF‐EGFR Binding Affinity

EGF proteins were coated on the plate wells with coating buffer at 4 °C overnight. EGFR containing His‐tag bound to EGF in a dose ladder (10‐3, 10–2, 10–1, 1, 10, 100 µm) with PEEPA‐P5 or Vehicle at 37 °C for 2 h and then incubated with His antibody containing horseradish peroxidase (HRP) at 37 °C for 1 h. HRP substrate Tetramethylbenzidine (TMB) was administrated into samples at 37 °C for 20 min and the OD value of each sample was measured by in Infinite Lumi (Tecan).

### Histopathology Scoring

For murine gastritis samples, histologic scoring was blindly done following standard criteria. In brief, the oxyntic atrophy scoring was determined by the absence of parietal and chief cells in mucosa and the gastritis histology index has three categories, inflammation, epithelial defects, and oxyntic atrophy. A larger score represents a more severe gastritis.

### Statistical Analysis

All data are displayed as the mean ± standard error of the mean (SEM) or standard deviation (SD). Unpaired, two‐sided, independent Student's *t*‐test (for two groups) and two‐way ANOVA using Bonferroni analysis (for more than two groups) were performed and are labelled in all figure legends. *p* < 0.05 indicates a statistically significant difference.

## Conflict of Interest

The authors declare no conflict of interest.

## Author Contributions

K.L., X.M. and Z.L. contributed equally to this work. Research design: B.L. and F.G., Murine assays: K.L., Z.L., Y.L., G.S., and D.W., Peptide screening: X.M., Cell line assays: Z.L. and Zc.L., Organoid culture: K.L. and Z.L., Mass spectrum: L.X., Human tissue sample analysis: Z.W. and M.T., Writing paper: H.L., F.G. and B.L.

## Supporting information

Supporting Information

## Data Availability

The data that support the findings of this study are available from the corresponding author upon reasonable request.
