# Supplementary material for: A Natural Peptide from A Traditional Chinese Medicine Has the Potential to Treat Chronic Atrophic Gastritis by Activating Gastric Stem Cells
Source: Adv Sci (Weinh). 2024 Mar 27;11(20):2304326. doi: 10.1002/advs.202304326 (PMC11132046; doi:10.1002/advs.202304326)
Supplement: Supplementary file 1 — Supporting Information [file ADVS-11-2304326-s001.pdf]

ADVANCED SCIENCE
Open Access

Supporting Information

for *Adv. Sci.*, DOI 10.1002/advs.202304326

A Natural Peptide from A Traditional Chinese Medicine Has the Potential to Treat Chronic Atrophic Gastritis by Activating Gastric Stem Cells

Ke Li, Xiuying Ma, Zihao Li, Ya Liu, Guiyan Shen, Zecheng Luo, Dong Wang, Li Xia, Zhengting Wang, Ming Tian, Huijuan Liu, Funeng Geng and Baojie Li**

Supporting Information for

A Natural Peptide from A Traditional Chinese Medicine Has the Potential to Treat Chronic Atrophic Gastritis by Activating Gastric Stem Cells

Ke Li[1,2], Xiuying Ma[3], Zihao Li[2], Ya Liu[1], Guiyan Shen[1], Zecheng Luo[1], Dong Wang[1], Li Xia[4], Zhengting Wang[5], Ming Tian[6], Huijuan Liu[2], Funeng Geng[3]*, Baojie Li[1,2]*
[1]Institute of Traditional Chinese Medicine and Stem Cell Research, College of Basic Medical Sciences, Chengdu University of Traditional Chinese Medicine, Chengdu, China.
[2]Bio-X Institutes, Shanghai Jiao Tong University, Shanghai 200240, China.
[3]Sichuan Good Doctor Panxi Pharmaceutical Co., Ltd., Chengdu 610000, China.
[4]Department of Pathophysiology, Key Laboratory of Cell Differentiation and Apoptosis of the Chinese Ministry of Education, Shanghai Jiao Tong University School of Medicine, Shanghai, 200025, China.
[5]Department of Gastroenterology, Ruijin Hospital, School of Medicine, Shanghai Jiao Tong University, Shanghai, China.
[6]Department of Burn, Ruijin Hospital, School of Medicine, Shanghai Jiao Tong University, Shanghai, China.
*Corresponding authors

Supplementary Experimental Section

Histology and immunostaining: Hematoxylin and eosin (H/E) staining and immunofluorescence staining were used to study the structure of gastric tissues. The stomach was harvested and fixed in 4% paraformaldehyde overnight. After dehydration in alcohol and embedment in paraffin, the tissue was sectioned longitudinally at 4 μm. The sections were stained with H/E following standard protocol. For immunofluorescence and immunohistochemistry staining, the tissue slides were boiled at 95°C in citrate buffer or EDTA buffer (pH 8.0) for antigen recovery, followed by cooling at room temperature. Slides were blocked by 10% goat serum for 30 min and incubated with primary antibodies at 4°C overnight. The primary antibodies include: Ki67 (Invitrogen, MA5-14520), PCNA (Santa Cruz, sc-56), GFP (CST, 2956), Gastrin (DAKO, A0568), TFF2 (ProteinTech, 13681), Muc5ac (Santa Cruz, sc-21701), H^+-K^+-

ATPase (Santa Cruz, sc-374094) and AQP5 (Santa Cruz, sc-514022). The sections were washed in PBS buffer and then incubated with second antibody at 37°C for 1h. The results were visualized in Nikon imaging system (ECILPSE, 80i).

Western Blot: Tissue proteins were isolated using T-PER Tissue Protein Extraction Reagent (Thermo Fisher, #78510). Proteins of cultured cells were extracted using RIPA lysis buffer supplemented with 1 mM PMSF, 1 mg/ml pepstatin, leupeptin, and aprotonin. Equal amounts of total proteins were subjected to SDS-PAGE and transferred to PVDF membrane (Millipore). The primary antibodies include: p-Stat1 (Invitrogen, 33-3400), Stat1 (CST, 14994), p-Stat3 (CST, 9145), Stat3 (CST, 9139), p-EGFR (CST, 3777), EGFR (CST, 4267), p-ERK1/2 (CST, 9106), ERK1/2 (CST, 9102), p-Smad1/5/8 (Millipore, AB3848), Smad1 (CST, 9743), β-Catenin (Santa Cruz, sc-7963), β-actin (Santa Cruz, sc-81178), p-Akt (CST, 4060), Akt (CST, 4691). The WB results were visualized and quantified using the FluoChem M system (ProteinSimple).

Cell culture and cell viability assay: Human gastric normal epithelial cell line GES-1 was cultured in DMEM medium containing 10% fetal bovine serum and 1% pencillin-streptomycin at 37°C with 5% CO2. For cell viability assay, Cell Counting Kit-8 (CCK-8) was purchased from KeyGEN BioTECH Corp., Ltd. The cells were cultured in 96-wells plate and PEEPA-P5 was added in each well with different concentrations. After 3 days, substrate tetrazolium salt was added to each sample and incubated at 37°C for 2 hrs and the absorbance at 450 nm was measured using BioTeck.

Quantitative PCR: Tissues or cells were lysed and the total RNA was isolated using the TRIzol reagent. cDNA was reverse transcribed from 500 ng RNA by PrimeScript RT Reagent Kit (TaKaRa, RR037A) and quantitative PCR was performed using Roche Light Cycler 480. The sequences of primers are as follows: Gastrin-F: 5'-GGACAGGGACCAATGAGG-3'; Gastrin-R: 5'-CCAAAGTCCATCCATCCGTAGG-3'; SST-F: 5'-ACCGGGAAAAGGAACTGG-3'; SST-R: 5'-TTGCTGGGTTCGAGTTGGC-3'; Muc5ac-F: 5'-GCCGTGTCAGGAGTCTAATACC-3'; Muc5ac-R: 5'-AGCCTAGCCACCACCTTAG-3'; TFF1-F: 5'-AGCACAAGGTGATCTGTGTCC-3'; TFF1-R: 5'-GGAAGCCACAATTTATCCTCTCC-3'; TFF2-F: 5'-TGCTCTGGTAGAGGGCGAG-3'; TFF2-R: 5'-CGAGCTAGAGTCAAAGAG-3'; Spdef-F: 5'-GGAGGAGACTCTTCTGAAG-3'; Spdef-R: 5'-GCTCCTGATGCTGCCTTCTCC-3'. For human genes, hPCNA-F: 5'-CCTGCTGGGATATTAGCTCCA-3'; hPCNA-R: 5'-CAGCGGTAGGTGTCGAAGC-3'; hTFF2-F: 5'-GCTGTTTCGACTCCAGTGTCA-3'; hTTF2-R: 5'-CCACAGTTTCTTCGGTCTGAG-3'; hMuc5ac-F: 5'-CAGCACAACCCCTGTTTCAAA-3'; hMuc5ac-R: 5'-GCGCACAGAGGATGACAGT-3'; hATP4b-F: 5'-TGGGTGTGGATCAGCCTGTA-3'; hATP4b-R: 5'-CTGGTCTTGGTAGTCCGGTG-3'; hGastrin-F: 5'-ATGCAGCGACTATGTGTGTATG-3'; hGastrin-R: 5'-GCCCCTGTACCTAAGGGTG-3'; hAxin2-F: 5'-TACACTCCTTATTGGGCGATCA-3'; hAxin2-R: 5'-TTGGCTACTCGTAAAGTTTTGGT-3'.

RNA sequencing: The gastric muscle layer was removed under the stereoscope and the

total RNA was isolated from epithelial cell samples (n=3 per group). After the quality control, cDNA was synthesized and amplified to DNA nanoball (DNBs). DNBs were measured by BGI-500 system (BGI-Shenzhen, China). Gene expression level was calculated and blasted with HISAT (v2.0.4) based on Burrows-Wheeler transform and Ferragina-Manzini. Clean reads were then detected in randomicity, coverage and degree of saturation. Statistically significant (p value = 0.05) genes with large expression changes (fold change R1.5) were defined as differentially expressed genes. In pathways analysis and Gene Ontology (GO) analysis, the candidate genes were assorted based on official classification with the KEGG or GO annotation result and phyper (a function of R) was performed in GO and pathway functional enrichment. Furthermore, GO analysis of stage-specific gene signature was analyzed in Biological Process and Cellular Composition. In addition, Gene Set Enrichment Analysis (GSEA) was performed using the GSEA_4.0.2 software (https://www.broadinstitute.org/gsea/) with metric = weighted, permutation = 1000, metric = Signal2Noise, permutation = gene set. All bulk RNA-seq datasets have been deposited in public database.

Single cell sequencing analysis: Raw reads data were downloaded, demultiplexed and mapped to the mouse or human reference genome by Cell Ranger version 3.0.1 (10x Genomics) pipeline with default parameters. The generated gene-cell express matrice was used for the subsequent analysis in R version 3.6.1 using Seurat version 3.1.5. The criteria of"Cells" as follow: <200 expressed genes, >25 UMIs mapped to mitochondria. Filtered cells were administrated to downstream graph-based clustering. Different expression genes (DEGs) in each cluster were identified with the function "FindAllMarkers" in Seurat. DEGs were mapped to the KEGG or GO database and the enrichment analysis of GO term or KEGG pathway was performed by the Clusterprofiler R package as previous described.

Immunoaffinity chromatography-tandem mass spectrometry: The active components (P5) in PEEPA were separated by absorption chromatograph column. Briefly, the column was incubated with EGFR antibody and the active peptides were bind to the column depended on the affinity between the antigen and antibody. Next, the active peptides were isolated by stripping buffer from the column and identified by mass spectrometry. The mixture of peptides was granulated into gas ion and separated according to different charge-to-mass ratios. The collective peptides were performed by blasting and sequencing to identify the molecular characters of substrates.

Identification of peptides via MS/MS ion spectra: The peptides analysis was performed on an Orbitrap Fusion LUMOS mass spectrometer (Thermo Fisher Scientific) connected to an Easy-nLC 1200 via an Easy Spray (Thermo Fisher Scientific). The peptides were loaded onto a self-packed analytical PicoFrit column with integrated spray tip (New Objective, Woburn, MA, USA) (75μm x 20cm length) packed with ReproSil-Pur 120A C18-AQ 1.9 μm (Dr. Maisch GmbH, Ammerbuch, Germany) and separated within a 60 minutes' linear gradient from 95% solvent A (0.1% formic acid / 2% acetonitrile / 98% water) to 28% solvent B (0.1% formic acid / 80% acetonitrile/ 20% water) at a flow rate of 300 nl/min at 50°C. The mass spectrometer was operated in positive ion mode and employed in the data-dependent mode within the specialized cycle time (2S) to automatically switch between MS and MS/MS. One full MS scan

from 350 to 1500 m/z was acquired at high resolution R=120,000 (defined at m/z=400); MS/MS scans were performed at a resolution of 30,000 with an isolation window of 1.6 Da and higher energy collisional dissociation (HCD) fragmentation with collision energy of 30±5%. Dynamic exclusion was set to 30s.

All MS/MS ion spectra were analyzed using PEAKS 10.6 (Bioinformatics Solutions) for processing, de novo sequencing and database searching. Resulting sequences were searched against the peptide 5（PAAEPVPLVKQD）with mass error tolerances of 10 ppm and 0.02 Da for parent and fragment, respectively, the digest mode specified as No digestion. FDR estimation was enabled. Peptides were filtered for −10log P ≥ 20 (P<0.01).

For concentration analysis of bioactive peptides, transitions generated by standard peptides were specified for P5-PAAEPVPLVKQD (Q1-632 Q3-699.2, 369.2, 895.4, 565.4, 390.2). All the extracted ion Chromatogram (XIC) were analyzed using PeakView 2.1.

Binding Affinity via surface plasmon resonance: The Avi-EGFR proteins were immobilized onto the surface of SA sensor chip with the immobilization of 600RU to calculate the K_D value of EGF protein. Briefly, EGF proteins were flowed over the EGFR surface. Then, the 10 mM glycine buffer (pH 3.0) was administrated for the regeneration of EGFR surface to remove the binding of EGF for 15s. Next, EGF proteins were diluted from 200 nM with a ladder of concentration and K_D value of EGF binding to EGFR was calculated by 10 different doses. For PEEPA-P5, immobilization of 5330RU was administrated and the regeneration time set at 150s. Furthermore, PEEPA-P5 was diluted from 100 μM.

Table S1. Blast search results for the PEEPA peptide (P5).

PEEPA-P5: Blast_P Results	Score (bits)	Query Cover (%)	E Value	Identities (%)	Gap (%)
Hypothetical protein [Pseudomonas sp. ADP]	35	100	1	75	25
Hypothetical protein [Pseudomonas humi]	35	100	1	75	25
Ig-like domain repeat protein [Pseudomonas delhiensis]	35	100	1	75	25
Hypothetical protein [Pseudomonas sp. EGD-AKN5]	35	100	1	75	25
Hypothetical protein SAMN05216189_106322, partial [Pseudomonas delhiensis]	35	100	1	75	25
Hypothetical protein [Pseudomonas citronellolis]	31.6	91	17	73.3	26
Type 1 secretion target domain-containing protein [Pseudomonas citronellolis]	31.6	91	17	73.3	26
Hypothetical protein AOQ84DRAFT_225504 [Glonium stellatum]	31.2	75	24	100	0

Table S2. Patient information.

No.	Sex	Age	Inflammation Score	Activity Score	Atrophy Score
U01927818	Male	58	+	-	+
D02879877	Female	66	+	-	+
D04095777	Male	59	+	-	++
C01241866	Female	63	+	+	+
G04219735	Female	63	+	-	++
A502604E6	Male	30	+	-	-
H509FF716	Female	26	+	-	-
C11027070	Male	27	+	-	-
P10330224	Female	43	+	-	-
L14606356	Female	30	+	-	-
G5010E1F3	Male	35	+	-	-
C03082037	Female	39	+	-	-
K02392859	Male	76	+	+	+
1320021011705	Male	66	++	+	++
B0179261X	Male	70	+	-	+
F02365977	Male	70	+	-	+
K01024536	Female	60	+	-	+
E01941904	Female	52	+	-	++

C0205072X	Male	64	+	-	+
U00530798	Male	67	+	-	++
B00981806	Female	NA	+	-	/
P05736828	Female	NA	+	-	-
C0055369X	Male	NA	+	-	/
M508D821X	Male	NA	+	-	-
A5023B8F3	Female	NA	+	-	-
B01412074	Female	NA	+	-	-
E00156736	Female	NA	+	-	+
132002102866752	Female	NA	+	-	-
B504406A4	Female	NA	+	-	+
P34341008	Male	NA	+	-	-
D18643674	Male	NA	+	-	-
D02439791	Male	NA	+	-	-
7100151650	Male	NA	+	-	/
D01954943	Male	NA	+	-	++
C00380514	Female	NA	+	-	+
A01469727	Male	NA	+	-	-
100001580219	Male	NA	+	-	-
A01470920	Female	NA	+	-	-
A50404AD6	Male	59	+	-	+
G01689088	Female	77	+	-	++
G04473995	Female	51	+	+	+
D0605352X	Male	53	+	-	+
CG1202061_330300	Male	76	+	-	+
YB1024011501048	Male	64	+	-	+
N01239280	Male	51	+	-	-
M50F33C89	Female	38	+	-	-
F12104201	Male	19	+	-	-
G05185215	Female	51	+	-	-
D04023474	Male	66	+	-	-
M5151C1D6	Male	38	+	-	-

Figure S1

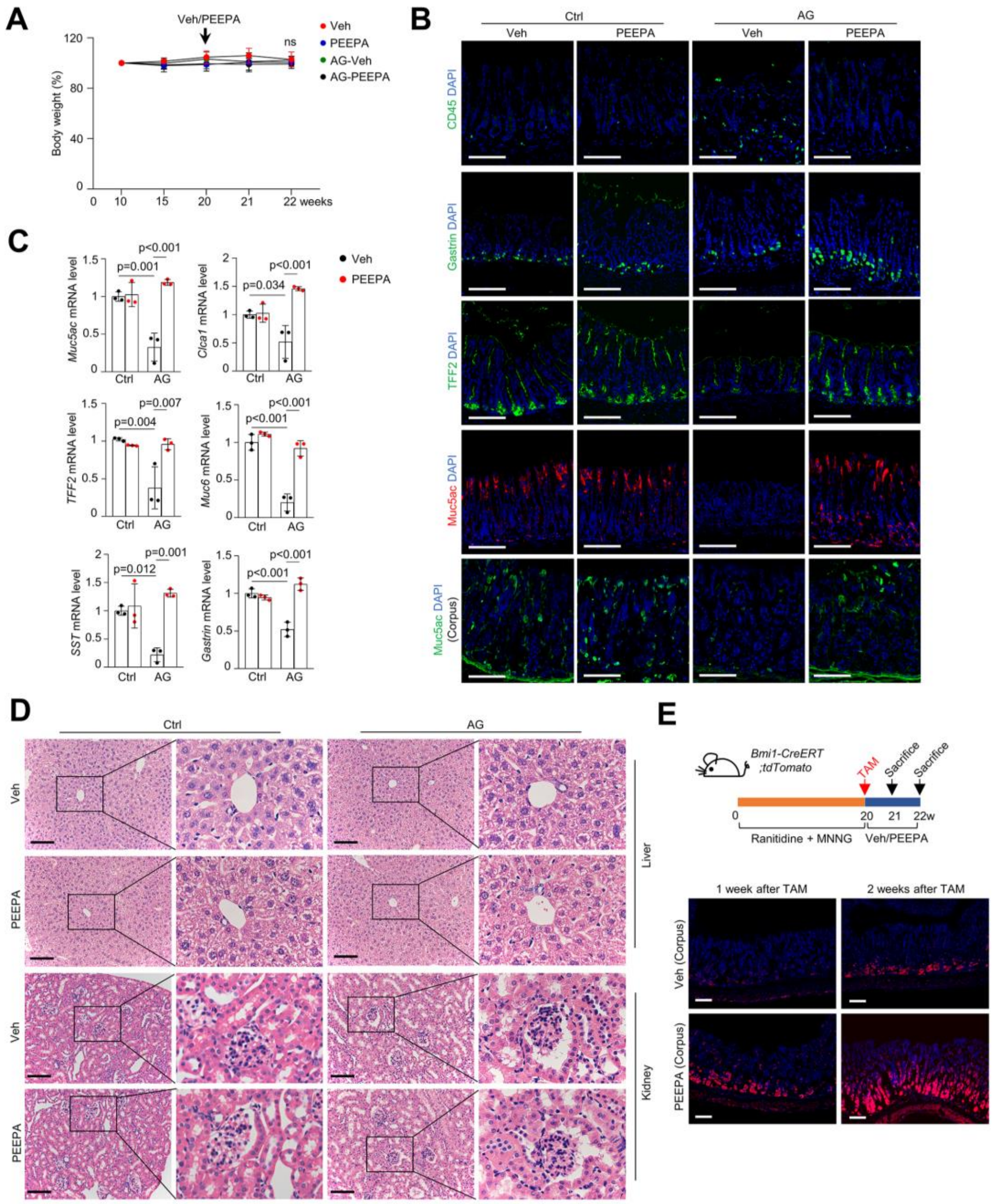

Figure S1 PEEPA has therapeutic effects on murine atrophic gastritis.

The change in body weight in AG model mice treated with PEEPA or vehicle (Veh). Data are the mean ± SD, n=5 per group.

Immunostaining revealed that PEEPA rescued the decrease in the numbers of cells expressing gastrin, TFF2, or Muc5ac and suppressed CD45^{+} cell infiltration in AG models.

qPCR results showed that PEEPA rescued the decrease in the expression of lineage-specific genes in the AG mice. *Muc5ac* and *Clca1* represent pit cells; *TFF2* and *Muc6* represent deep mucous cells; *SST* and *gastrin* represent endocrine cells. Data are the mean ± SD, n=3 per group.

Histology of the liver and kidney of AG model mice treated with Veh or PEEPA. H&E

staining images are representative of at least three mice for each group.

Tracing Bmi1+ GSCs and daughter cells in the isthmus and base of *Bmi1-CreERT; tdTomato* mice in the corpus. Upper panel: The schedule of TAM administration. AG mice were administered Veh or PEEPA for 1 or 2 weeks after 3 doses of TAM (10 mg/kg).

Two-way ANOVA was applied for (A) and (C). p<0.05 was considered statistically significant. Scale bars: 100 μm.

Figure S2

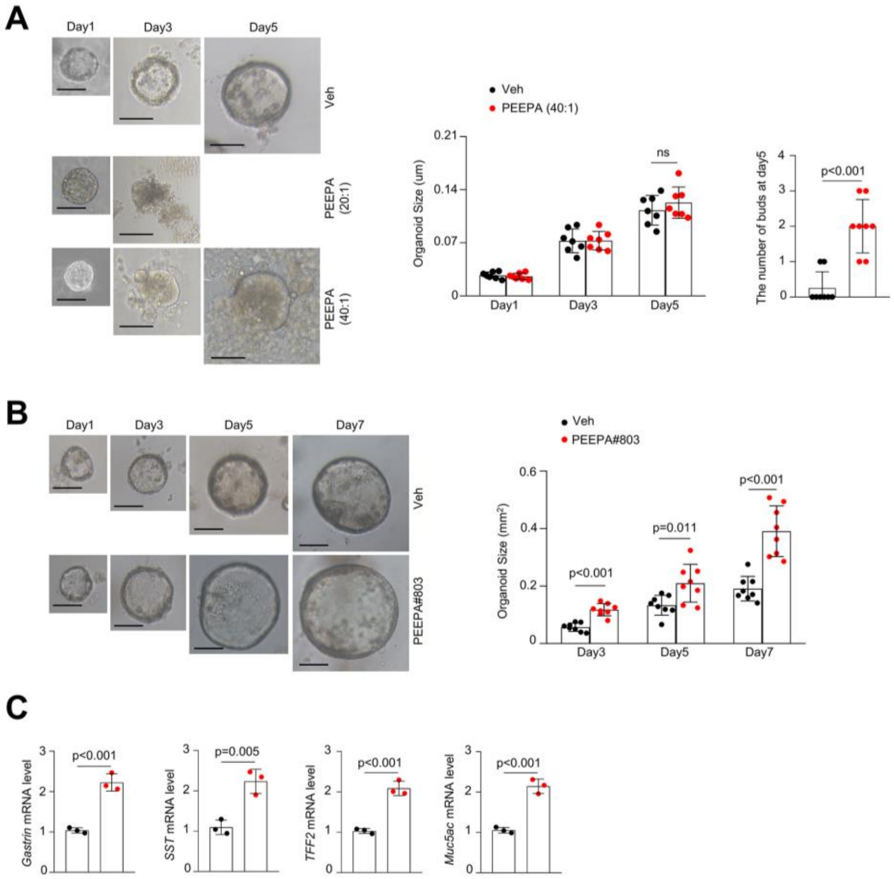

Figure S2 The effects of different doses or batches of PEEPA on gastric organoids.

Consecutive images showed the effects of high concentrations of PEEPA on gastric organoid growth. Right panel: quantitative data. Data are the mean ± SD, n=3 per group.

Consecutive images showed the effects of another batch of PEEPA (#803) on gastric organoid growth. Right panel: quantitative data. Data are the mean ± SD, n=3 per group.

qPCR results showed the effects of another batch of PEEPA (#803) on gastric organoid differentiation. Data are the mean ± SD, n=3 per group.

Unpaired two-tailed Student's t test was applied for (A), (B) and (C). p<0.05 was considered statistically significant. Scale bars: 100 μm.

Figure S3

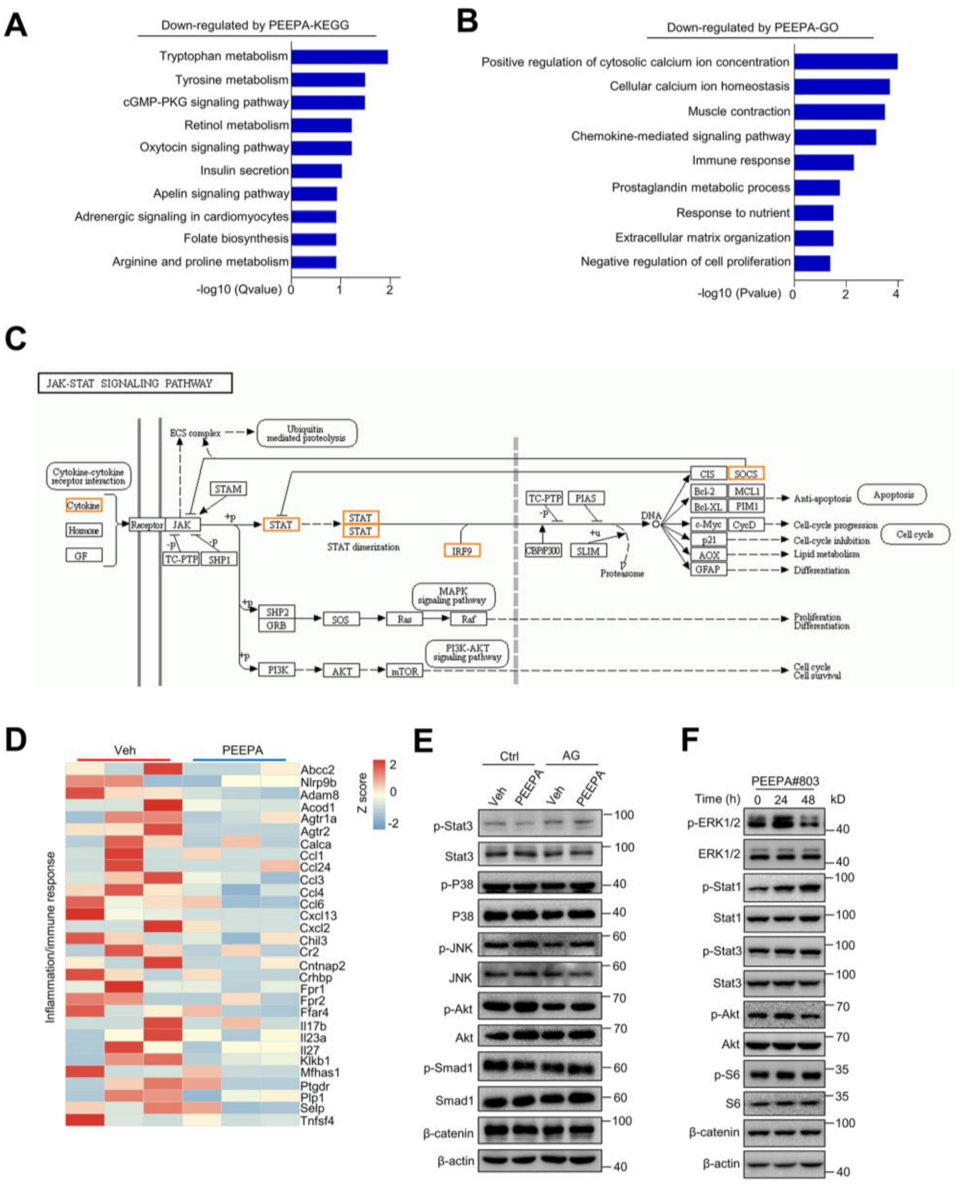

Figure S3 RNA-seq results showed the transcriptomes of gastric samples of mice treated with PEEPA or Veh.

KEGG analysis showed the downregulated signalling pathways in PEEPA-treated samples compared to control samples.

GO analyses showed downregulated biological modules in the PEEPA-treated group compared to the Veh-treated group.

Heatmap of the Jak-Stat pathway. The orange rectangle marks signalling molecules activated by PEEPA.

Heatmap results showing the expression of immune response-related genes in PEEPA-treated samples compared to control samples.

Western blot results showed no activation of Stat3, p38, JNK, Akt, or Smad1 by PEEPA

in a murine gastritis model.

Western blot results showed that another batch of PEEPA (#803) activated the EGFR downstream signalling pathways.

Figure S4

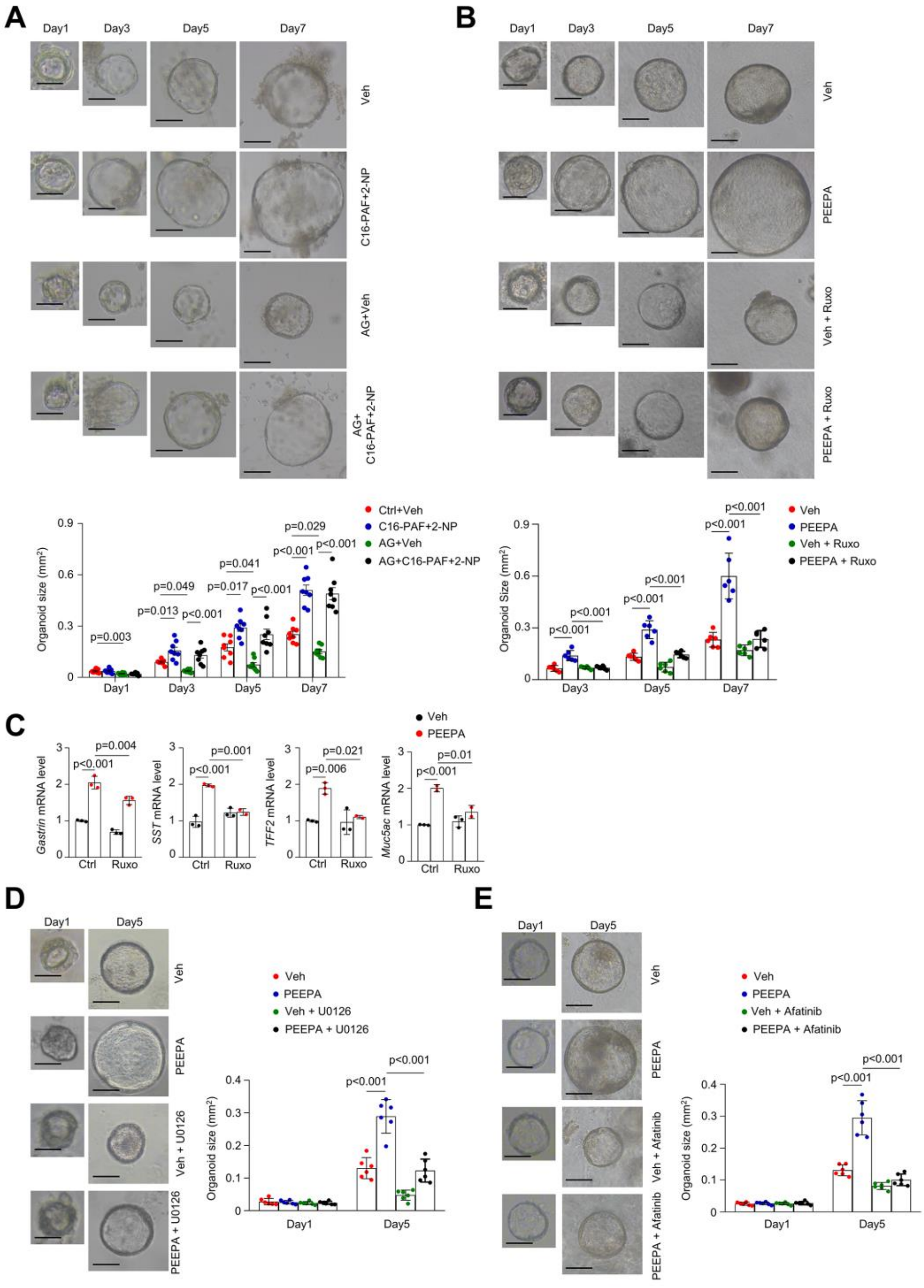

Figure S4 Inhibition of EGFR downstream signalling suppressed organoid growth. Representative organoid images showed that combined Stat1 and ERK agonists substituted EGF in organoid cultures. C16-PAF and 2-NP was added at a concentration of 1μM and 45 μM, respectively. Bottom panel: Quantitative data. Data are the mean ± SD, n=8 per group.
Representative organoid images showed that inhibition of Jak signalling impeded PEEPA-induced organoid growth. Ruxolitinib (Ruxo) was administered at a concentration of 15 μM. Right panel: Quantitative data. Data are the mean ± SD, n=6 per group.
qPCR results showed that Ruxo suppressed the expression of differentiation markers in gastric organoids. Data are the mean ± SD, n=3 per group.
Representative organoid images showed that inhibition of ERK signalling impeded PEEPA-induced organoid growth. U0126 was administered at a concentration of 20 μM. Right panels: quantitative data. Data are the mean ± SD, n=3 per group.
Representative organoid images showed that inhibition of EGFR signalling impeded PEEPA-induced organoid growth. Afatinib was administered at a concentration of 1 μM. Right panels: quantitative data. Data are the mean ± SD, n=3 per group.
Two-way ANOVA was applied for (A), (B), (C), (D)and (E). $p<0.05$ was considered statistically significant. Scale bars: 100 μm.

Figure S5

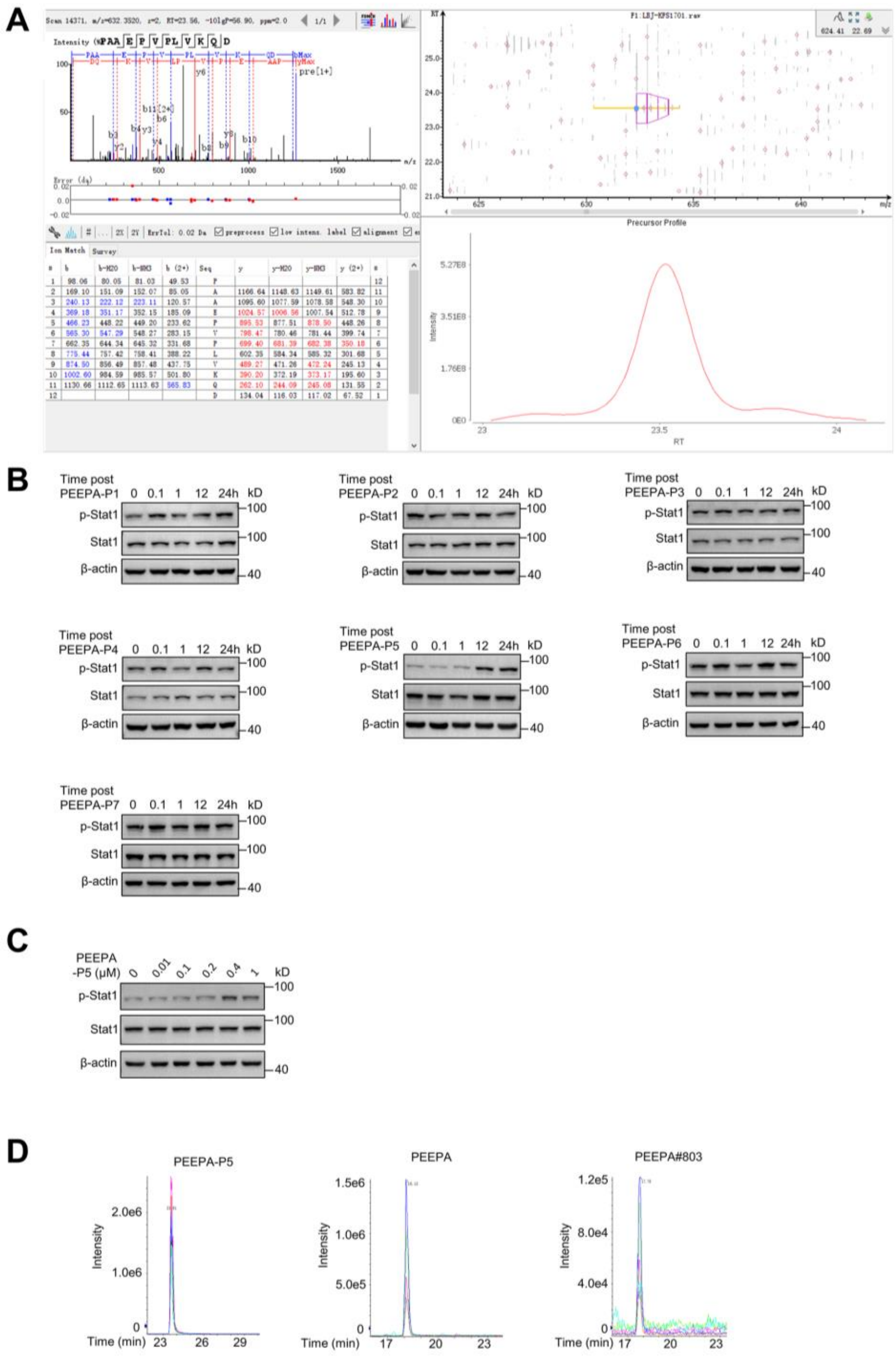

Figure S5 Analysis of the PEEPA peptides.

Mass spectrometry analysis of the PEEPA-P5 peptide sequence from PEEPA. The mass spectrometer was operated in positive ion mode and employed in the data-dependent mode within the specialized cycle time (2S) to automatically switch between MS and MS/MS. One full MS scan from 350 to 1500 m/z was acquired at high resolution R=120,000 (defined at m/z=400); MS/MS scans were performed at a resolution of 30,000 with an isolation window of 1.6 Da and higher energy collisional dissociation (HCD) fragmentation with collision energy of 30% +/- 5. Dynamic exclusion was set to 30 s.

Representative western blot results showed that PEEPA-P5 was the most potent in the activation of Stat1 in GES-1 cells.
Western blot results showed that PEEPA-P5 activated p-Stat1 at 0.4 μM in GES-1 cells.
Mass spectrometry detection of the PEEPA-P5 peptide in the different batches of PEEPA. The peptide analysis was performed on the 6500plus (ABsciex) connected to the Capillary-LC eksigent415. Transitions generated by standard peptides were specified for PEEPA-P5-PAAEPVPLVKQD (Q1-632 Q3-699.2, 369.2, 895.4, 565.4, 390.2). The mass spectrometry was operated in positive ion mode and performed in MRM mode.

Figure S6

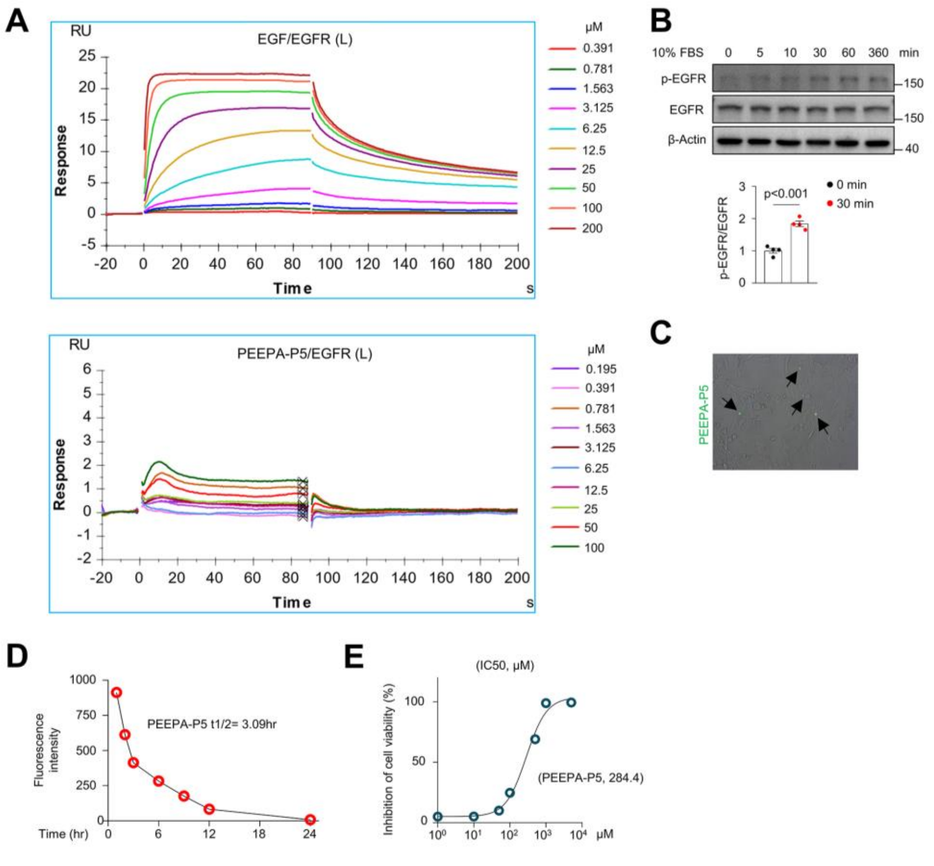

Figure S6 Analysis of the major features of PEEPA-P5.
Determination of the affinity of PEEPA-P5 to EGFR (lower). HBS-P running buffer was administered at a 30 μl/min flow rate for 60 seconds.
Representative Western blot results showed that fetal bovine serum activated EGFR in GES-1 cells after 24-hour starvation. Bottom panel: quantitation data for time 0 and 30 min (when activation reached the maximal). Data are the mean ± SD, n=4 per group.
The location of PEEPA-P5-FITC in GES-1 cells. The arrows showed signals on the surface of cells.
The retention time of PEEPA-P5-FITC in the murine stomach. The fluorescence of

PEEPA-P5 was measured at different time points after oral gavage.
The IC_{50} assay revealed low cell toxicity of PEEPA-P5 towards GES-1 cells. Data are the mean ± SD, n=3 per group.
Unpaired two-tailed Student's t test was applied in (E). Two-way ANOVA was applied for (B). $p<0.05$ was considered statistically significant.

Figure S7

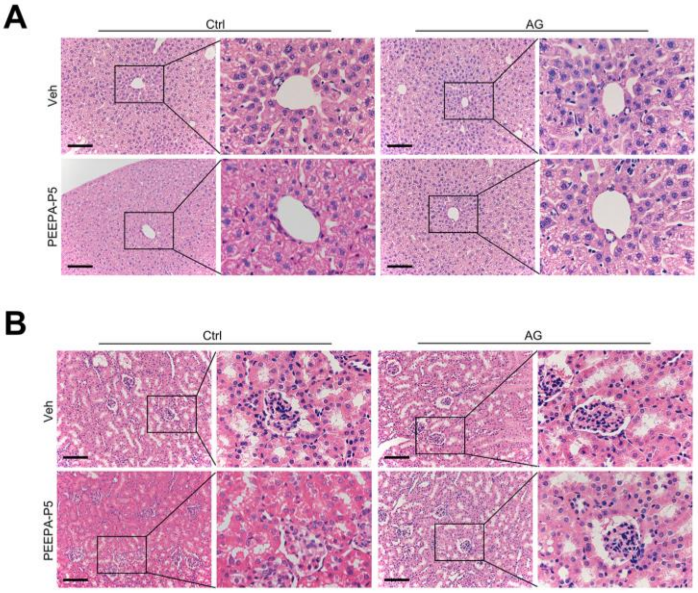

Figure S7 PEEPA-P5 has little effect on the kidney or liver structure.
H&E staining showed that liver structure was not altered by PEEPA-P5. Images of H&E staining are representative of three mice for each group.
H&E staining showed that kidney structure was not altered by PEEPA-P5. Images of H&E staining are representative of three mice for each group. Scale bars: 100 μm.

Figure S8

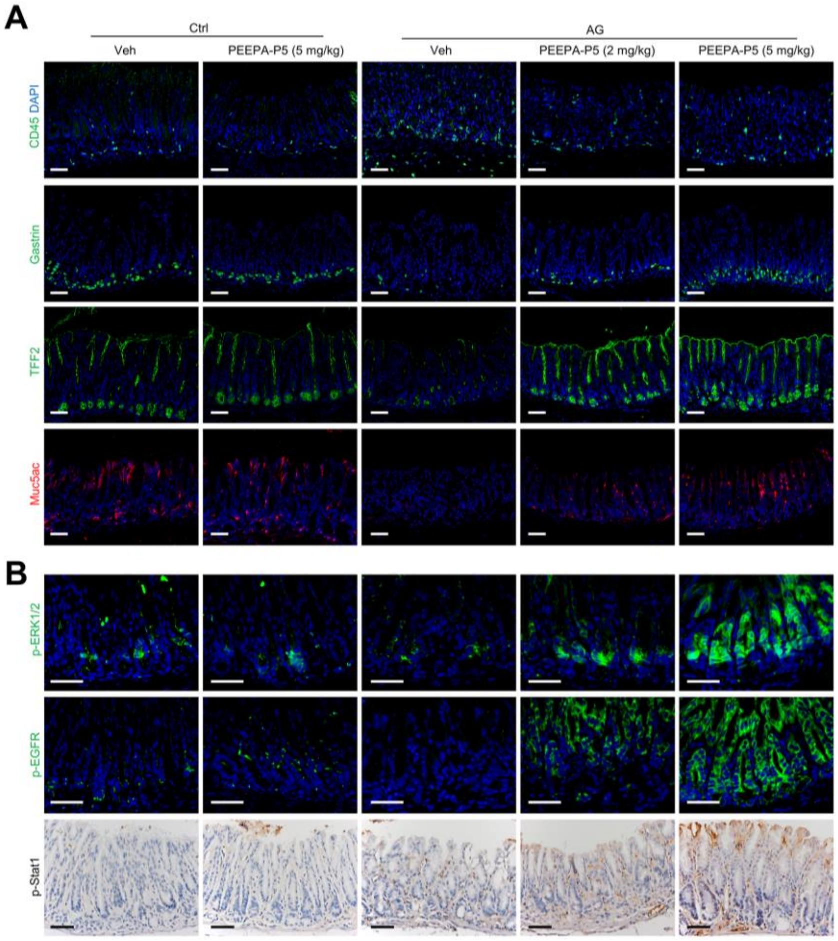

Figure S8 PEEPA-P5 rescues the differentiation defects and activates EGFR-Stat1 signalling in AG models.

Immunostaining results showed that PEEPA-P5 rescued the decrease in the numbers of cells expressing gastrin, TFF2 or Muc5ac and suppressed CD45^{+} cell infiltration in AG model mice.

Representative immunostaining results showed the activation of EGFR, Stat1, and ERK1/2 in stomach sections of PEEPA-P5-treated AG mice. Scale bars: 100 μm.

Figure S9

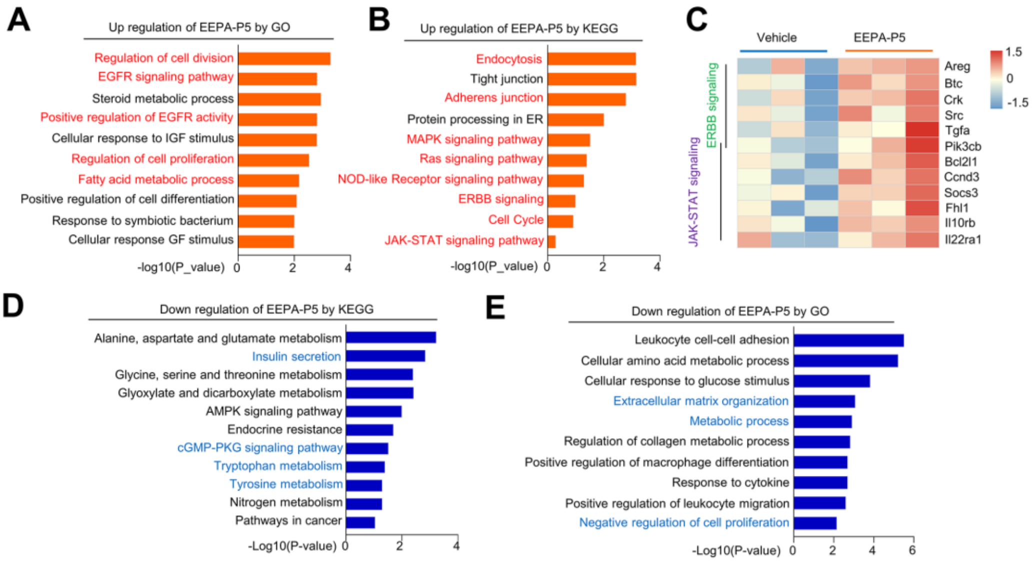

Figure S9 Bulk RNA sequencing analysis of P5-treated mouse samples.

A, B. GO (A) and KEGG pathway (B) analyses detected upregulated signalling pathways and modules in atrium samples of P5-treated compared to vehicle-treated AG mice. The signalling pathways and modules that were also up-regulated by PEEPA were marked in red.

C. Heatmap results showed the alteration in the expression of the ERBB signalling and Jak-Stat signalling pathway genes enhanced by P5 treatment.

D, E. KEGG pathway (D) and GO (E) analyses uncovered down-regulated signalling pathways and modules in P5-treated compared to vehicle-treated AG mice. The signalling pathways and modules that were also up-regulated by PEEPA were marked in blue.

Figure S10

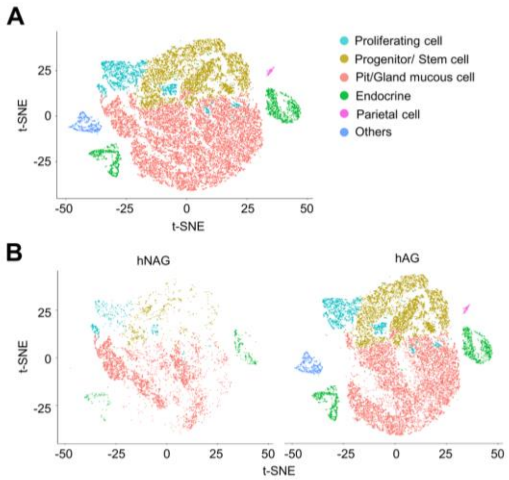

C

Cell types (%)	Gastrin+	Muc5ac+	TFF2+	CDK1+	PCNA+	CCK2R+
hNAG	85.43	92.96	92.58	3.52	12.69	1.61
hAG	53.98	72.62	71.76	2.86	10.25	1.14

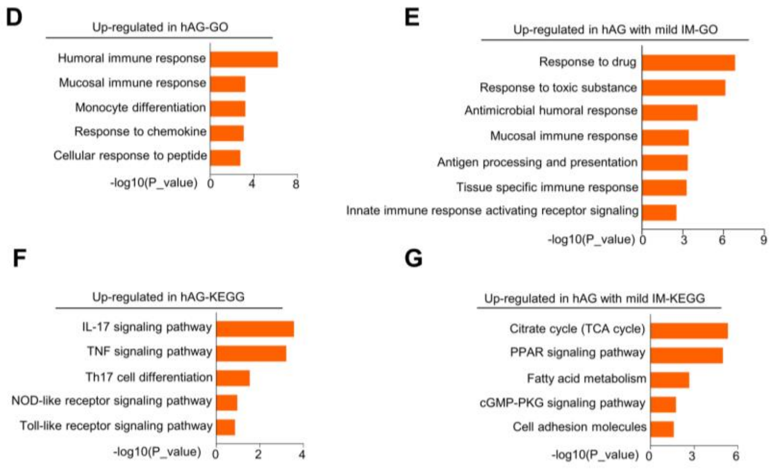

Figure S10 Analysis of single-cell RNA sequencing data of human AG samples.
Clustering of the cell types in NAG and AG samples.
Comparison of the clustering of the cell types in NAG and AG patient samples (36,809 cells for total AG and 6134 cells for NAG).
Quantitative results showed a decline in the percentage of endocrine cells (gastrin^{+}), pit cells (Muc5ac^{+}), gland mucous cells (TFF2^{+}), proliferating cells (CDK1^{+} and PCNA^{+}) and stem cells (CCK2R^{+}) in AG samples compared to NAG samples (36,809 cells for AG and 6134 cells for NAG).
KEGG pathway analysis revealed the upregulation of signalling in AG compared to NAG from scRNA-seq data. Three NAG biopsies and three AG biopsies were used for this analysis.
GO analysis showed the upregulation of biological processes in AG samples compared to NAG samples.
KEGG pathway analysis detected the upregulation of signalling pathways in AG with mild intestinal metaplasia (IM) compared to NAG samples. Three NAG biopsies and

two AG with mild IM biopsies were used for this analysis.
GO analysis showed upregulation of biological processes in AG with mild intestinal metaplasia (IM) compared to NAG patient samples.
